# Cross-species investigation into the requirement of XPA for nucleotide excision repair

**DOI:** 10.1093/nar/gkad1104

**Published:** 2023-11-22

**Authors:** Cansu Kose, Xuemei Cao, Evan B Dewey, Mustafa Malkoç, Ogün Adebali, Jeff Sekelsky, Laura A Lindsey-Boltz, Aziz Sancar

**Affiliations:** Department of Biochemistry and Biophysics, University of North Carolina School of Medicine, Chapel Hill, NC, USA; Department of Biochemistry and Biophysics, University of North Carolina School of Medicine, Chapel Hill, NC, USA; Department of Biology, University of North Carolina at Chapel Hill, Chapel Hill, NC, USA; Faculty of Engineering and Natural Sciences, Sabanci University, Istanbul, Türkiye; Faculty of Engineering and Natural Sciences, Sabanci University, Istanbul, Türkiye; Department of Computational Science-Biological Sciences, TÜBITAK Research Institute for Fundamental Sciences, Gebze, Türkiye; Department of Biology, University of North Carolina at Chapel Hill, Chapel Hill, NC, USA; Department of Biochemistry and Biophysics, University of North Carolina School of Medicine, Chapel Hill, NC, USA; Department of Biochemistry and Biophysics, University of North Carolina School of Medicine, Chapel Hill, NC, USA

## Abstract

After reconstitution of nucleotide excision repair (excision repair) with XPA, RPA, XPC, TFIIH, XPF-ERCC1 and XPG, it was concluded that these six factors are the minimal essential components of the excision repair machinery. All six factors are highly conserved across diverse organisms spanning yeast to humans, yet no identifiable homolog of the *XPA* gene exists in many eukaryotes including green plants. Nevertheless, excision repair is reported to be robust in the XPA-lacking organism, *Arabidopsis thaliana*, which raises a fundamental question of whether excision repair could occur without XPA in other organisms. Here, we performed a phylogenetic analysis of XPA across all species with annotated genomes and then quantitatively measured excision repair in the absence of XPA using the sensitive whole-genome qXR-Seq method in human cell lines and two model organisms, *Caenorhabditis elegans* and *Drosophila melanogaster*. We find that although the absence of XPA results in inefficient excision repair and UV-sensitivity in humans, flies, and worms, excision repair of UV-induced DNA damage is detectable over background. These studies have yielded a significant discovery regarding the evolution of XPA protein and its mechanistic role in nucleotide excision repair.

## Graphical Abstract

**Graphical Abstract:**
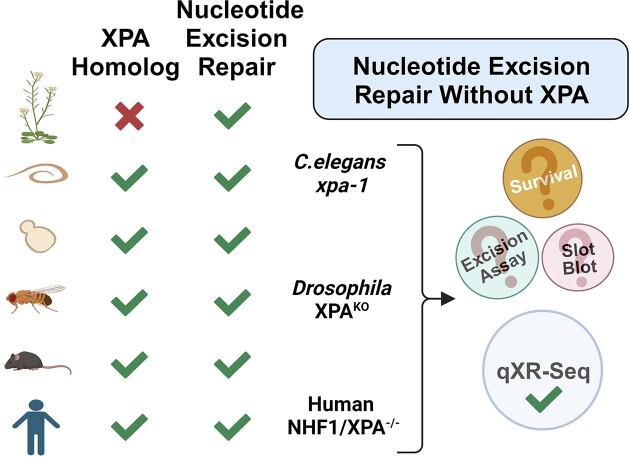

## Introduction

As genome integrity is critical for life, organisms have evolved complex repair pathways to target the various forms of DNA damage induced by endogenous and exogenous events. Among these, nucleotide excision repair is responsible for repairing a wide range of helix-distorting DNA lesions, including the UV-induced photoproducts cyclobutane pyrimidine dimers (CPDs) and 6–4 pyrimidine-pyrimidone photoproducts [6–4)PPs]. Many of the key factors required for excision repair, XPA-XPG, were originally identified by complementation assays using cell lines derived from patients with a hereditary condition called Xeroderma Pigmentosum (XP) which is characterized by increased sensitivity to sunlight and high incidence of skin cancers (1,2). Subsequently, it was determined that XPA, XPB, XPC, XPD, XPF and XPG are essential components of the excision repair machinery (3–8) and highly conserved across diverse organisms spanning yeast, worms, fruit flies, and humans (9–13). In fact, it has been demonstrated that the *Drosophila melanogaster* XPA (dmXPA) functionally complements the UV-sensitivity of a human XP-A patient cell line lacking hXPA (14). Nevertheless, although XPA has been shown to be an important factor in the DNA damage recognition and scaffolding interactions required for efficient excision repair in many eukaryotes (4,15), there are no identified homologs of the *XPA* gene in the genomes of some eukaryotes (16). Here, we performed an exhaustive phylogenetic analysis to infer the evolutionary history of the *XPA* gene across all eukaryotes and were able to assign multiple gene duplication and deletion events. With a couple of exceptions due to horizontal gene transfer, *XPA* is found in none of the plant species, including green plants such as *Arabidopsis* and green algae, and red algae. Moreover, although *XPC* homologs are present in Discoba, the species in this clade including *Trypanosoma* lack *XPA*.

Since excision repair is robust in the XPA-lacking green plant *Arabidopsis thaliana* (17–19), we wished to determine whether excision repair could also occur in other organisms in the absence of XPA. We recently developed a very sensitive method which directly captures and identifies the excised oligomers to measure repair throughout the genome in a quantitative manner, named qXR-Seq for quantitative eXcision Repair-Sequencing, with which we were able to observe extremely low levels of repair in human cell lines lacking the XPC and CSB excision repair factors (20). Here we use this method to investigate excision repair in the absence XPA in two human fibroblast cell lines and in two widely studied model organisms, *D. melanogaster* and *Caenorhabditis elegans*. We found that although both human *XPA^−/−^* cell lines that we generated are extremely UV sensitive and have essentially undetectable repair activity by the slot blot assay, in agreement with previous reports (21,22), we were able to detect UV-dependent oligonucleotides with properties consistent with excision by nucleotide excision repair (appropriate location of dipyrimidine and preferential repair of the transcribed strand of genes). Although the relative excision repair in the *XPA^−/−^* human cells is approximately 4-fold over background (the no UV condition from the same cell line), it is only ∼0.001% relative to the amount of excision repair observed in wildtype cells.

Since it was somewhat unexpected to observe excision repair in *XPA^−/−^* human cell lines, we wished to determine whether excision repair in the absence of XPA could be detected in other model organisms. We obtained a *C. elegans xpa-1* mutant strain known to be extremely UV-sensitive (23–25) and excision repair-deficient as determined by several different methods: enzyme-sensitive site assay (26), qPCR assay (27), and slot-blot assay (28). Surprisingly, in worms we also observed excision repair approximately 10-fold over background in the XPA mutant, and approximately 0.003% excision repair in the *xpa-1* worms relative to wildtype worms. Finally, since we had previously examined excision repair in *Drosophila melanogaster* and found that it has some interesting mechanistic similarities and differences to many other organisms previously studied (29,30), we decided to employ this model organism as well. There are no documented reports of UV- or drug-sensitive phenotypes associated with the *dmXPA* gene, so we generated XPA knockout flies (XPA^KO^). Surprisingly, when we compared the newly generated XPA^KO^ flies to our previously generated XPC^KO^ flies lacking detectable excision repair activity (29) we found that the XPA^KO^ flies are not as UV-sensitive. In agreement with this observation, results from our qXR-Seq experiments revealed extensive excision repair in the XPA^KO^ flies (100-fold over background and ∼1% repair relative to wildtype flies). Taken together, we conclude that although it is inefficient, excision repair of UV-induced DNA damage does occur in humans, worms, and flies in the absence of XPA.

## Materials and methods

### Phylogenetic analysis

#### Reference proteomes and homology search parameters

We obtained 2224 eukaryotic reference proteomes from The UniProt (31) proteomes database (release-2023_02) (https://ftp.uniprot.org/pub/databases/uniprot/previous_releases/release-2023_02/knowledgebase) on 3 March 2023. Additionally, we acquired sequences of human repair proteins XPA (P23025), XPC (Q01831), XPF (Q92889), XPG (P28715) and ERCC1 (Q1LZ75) from The UniProtKB database (31). We conducted a homology search using two steps. Firstly, we utilized the *blasp* option of the BLAST algorithm (32) with a repair protein as the query against a primary BLAST database derived from the eukaryotic proteomes that we created as a local database. Since reconstructing maximum likelihood trees with many sequences can be costly, we chose to limit our sequence number by selecting homologous sequences using BLAST hits. We stopped retrieving sequences after acquiring three homologs of the human repair protein. Including at least one human protein is important because we use paralogs as outgroups in the tree rooting step. In the case of XPA homologs, we only obtained one additional human protein (ZNT9), so we retrieved all hits from that BLAST output. For each of the five repair proteins, we used the BLAST hits to construct a multiple sequence alignment (MSA) using the fftns option from the MAFFT v7.490 algorithm (33), specifically recommended for > 2000 sequences. Before tree construction, we used trimAl tool with *gappyout* option in order to exclude redundant gaps in MSA. After the trimming, for specifying the substitution model that is needed for building phylogeny, IQTree algoritm's (34) *modelfinder* was used and RAxML-NG v1.0.3 tool (35) was utilized with ‘LG4X model’ with default parameters. For the trees, we initially applied midpoint rooting. The reason why we constructed phylogenetic trees was to determine the functional homologs of the protein in question. We benefitted from the second hit for XPA to decide where to place the tree root. After placing the roots manually, we added taxonomic lineage information to the nodes and identified the duplication nodes with custom scripts (github.com/CompGenomeLab/XPA_evolution) benefiting from the ETE3 toolkit (36). With the added information, we investigated the trees in FigTree v1.4.4 (http://tree.bio.ed.ac.uk/software/figtree/).

#### Elimination of non-XPA homologous sequences

We applied three techniques requiring manual curation. First, we identified paralogs by considering the species distribution in the two sister clades. The XPA homologous clade emerging from the root of the tree was removed. This clade involved a human zinc transporter protein. Secondly, we considered the domain architecture of the homologous proteins. To eliminate remote homologs, we discarded the sequences with significant divergence from XPA with respect to the domains they contained. For this purpose, we ran *hmmscan* of HMMER software (37) against Pfam-A 32.0 database (38). Finally, we manually analyzed the MSA of the XPA, with Jalview (39) in detail, and came up with highly conserved residues, which can be used as a ‘signature’ to decide on the homologs that are likely preserving the XPA function. Once we completed the necessary steps, we verified that the MSA consisted of XPA homologs that were functionally equivalent. However, since it is possible that we did not include all relevant sequences, we conducted a more sensitive homology search utilizing PSI-BLAST by inputting our MSA. After incorporating the additional sequences that were discovered, we repeated the same three steps only using FastTree v2.1.10 (40) for the phylogeny construction due to its speed.

#### Species tree construction

**Figure 1.**
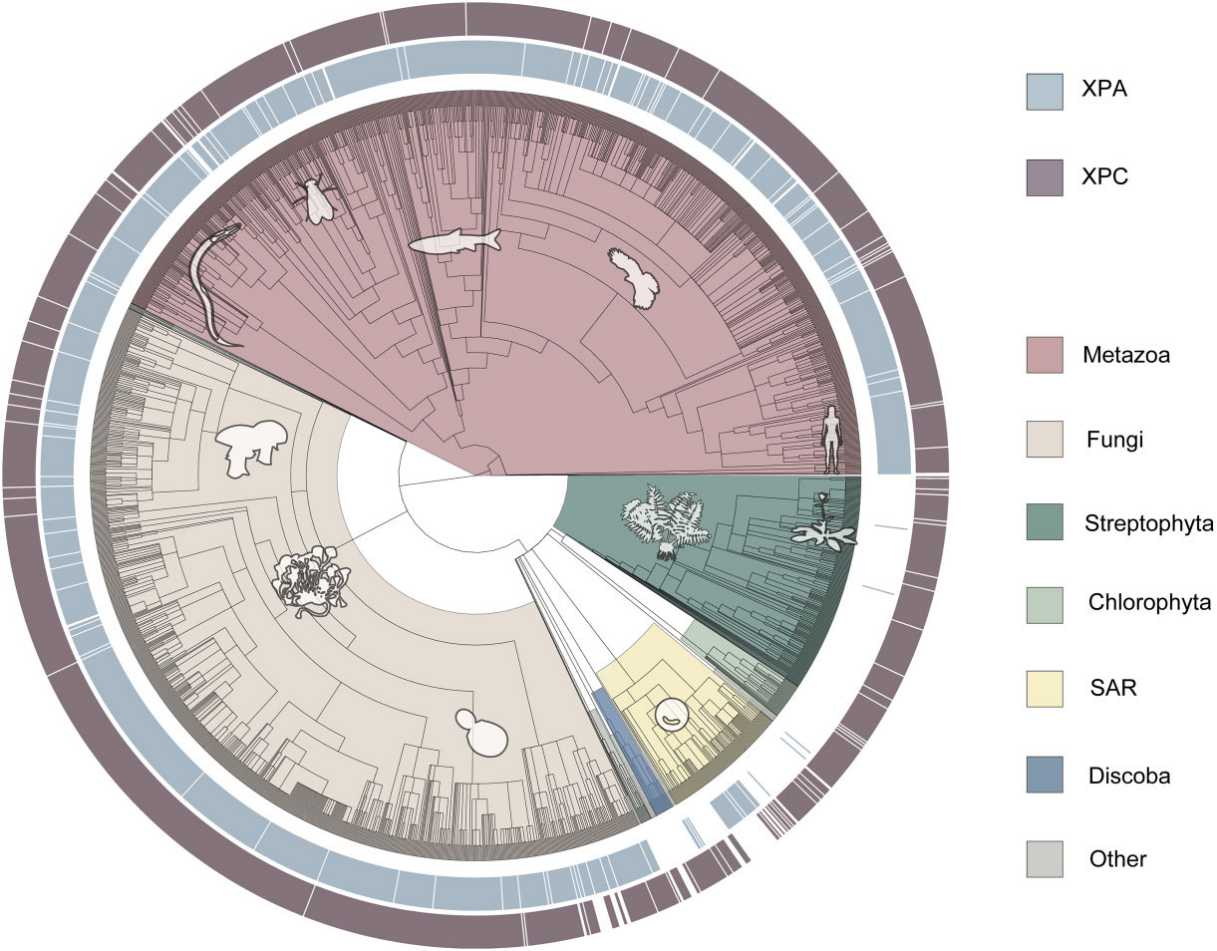
Phylogenetic tree indicating the presence of the *XPA* (light blue) and *XPC* (mauve) genes in the sequenced species of the indicated clades of life. The *XPC* gene is included as a control, as species lacking the genes (white) of both excision repair factors are likely to have incomplete genomes. *XPA* is not found in most species of the Discoba clade, which includes *Trypanosoma*, the Viridiplantae kingdom of green plants, which includes *Arabidopsis*. For the complete list of analyzed species see Supplementary Table S1.

We utilized the NCBI Taxonomy Browser's CommonTree tool (41) found at https://www.ncbi.nlm.nih.gov/Taxonomy/CommonTree/wwwcmt.cgi. We gathered the taxonomy IDs from our proteomes and utilized them to develop the tree. While constructing the tree, we opted for the unranked taxa option. CommonTree provides a representative common tree in phylip format; however, we had to modify the topology of the tree for a better representation. Unfortunately, the phylip format provided by CommonTree was unsuitable for parsing with ETE. Therefore, we converted it to newick format. Besides this, CommonTree does not provide some of the species from our list in the initial phylip tree. Even though not many of them seemed to have an XPA homolog, some of them (43 proteins from 41 species) were in the XPA tree and we excluded them too for a better display of our results. During our analysis, we discovered some species with none of the five repair proteins we investigated. We believe this is due to incomplete or low-quality proteomes. Consequently, we excluded these species from our analysis. After constructing the species tree, we proceeded with our analysis using 2132 species (Supplementary Table S1) and have displayed the presence of XPA across species (Figure 1), using XPC as a control group due to its high conservation and central role in global repair.

### Biological resources

The wildtype normal human skin fibroblast (NHF1/WT) and NHF1/XPC^−/−^/CSB^−/−^/XPA^−/−^ cell lines have been previously described (42,43). Clustered Regularly Interspaced Short Palindromic Repeats (CRISPR)-Cas9 technology was used to generate the mutant NHF1/XPA^−/−^ cell line (Supplementary Figure S2A). Single clones were isolated and successful knockout (KO) was confirmed by sequencing genomic DNA (Supplementary Figure S2B) and by a lack of XPA protein by western blot analysis (Supplementary Figure S2C). The *C. elegans* wild-type (N2 ancestral) and *xpa-1 (ok698)* strain was obtained from the *Caenorhabditis* Genetics Center and were cultured under standard conditions at room temperature on nematode growth media plates with *E. coli* strain OP50. The *D. melanogaster XPA* gene was knocked out using CRISPR-Cas9 technology (Supplementary Figure S3) as described previously for *XPC* (29) with the following changes: The endogenous *XPA* gene was deleted and replaced with dsRed using CRISPR/Cas9 genome engineering and scarless allele replacement similar to that described previously (44). A plasmid containing dsRed under the control of an 3XP3 (eye expression) promoter and flanked with DNA homologous to 5′ and 3′ *XPA* flanking sequence (pGEM XPA 5′+3′ Donor) and another plasmid containing 5′ and 3′ XPA gRNAs were (pCFD4 XPA gRNA) were simultaneously injected into *Drosophila* embryos expressing Cas9 in their germline stem cells under control of the *nanos* promoter (Genetivision, Houston, TX). Male progeny were screened for expression of dsRed (and likely deletion of *XPA*) in their eyes using a fluorescent microscope. These males were then mated to *FM7w* females to isolate and balance the suspected deletion. Deletions were then further screened via genomic extraction, PCR, and sequencing of parental flies used to establish the *XPA* deletion stock to confirm the deletion. All parents contained both the correct 5′ and 3′ flanking genomic sequence, indicating that *XPA* was successfully deleted and replaced with dsRed.

### Survival, slot-blot and qXR-Seq assays

Survival and slot blot assay procedures have been described previously (29,45). For the human qXR-Seq experiments, cells were harvested 2h after treatment with 20J/m^2^ of UVC and qXR-Seq was performed as previously described (20). Two thousand times more starting material was used for the *XPA^−/−^* cell lines than for wildtype NHF1 and qXR-Seq was conducted by adding UV-irradiated *Drosophila* Hirt lysate as previously described (20). For *Drosophila* qXR-Seq experiments, *Drosophila* were harvested 2 h after treatment with 1200 J/m^2^ of UVB and XR-Seq was performed similarly as previously described (20) with the following modifications for quantitation. After Hirt lysis, different dilutions of the wildtype sample were made from 1 to 500, and the same amount (2000-fold less compared to *Drosophila*) of UV-irradiated *C. elegans* Hirt lysate was added to each. For the *C. elegans* qXR-Seq experiments, *C. elegans* were cultured on 150 mm plates and exposed to 4000 J/m^2^ of UVB radiation. One hour after the treatment, the animals were collected in M9 buffer and washed until the supernatant became clear. The pelleted *C. elegans* were then incubated for 2 h at 62°C with 450 μl of Worm Hirt Lysis Buffer (0.15M Tris pH 8.5, 0.1M NaCl, 5mM EDTA, 1% SDS) and 20 μl of Proteinase K (NEB, cat. no. P8107S). Subsequently, 120 μl of 5M NaCl was added, and the mixture was inverted to ensure proper mixing, followed by an overnight incubation and one hour centrifugation at 4°C. Wild type worm samples were serially diluted from 1, 1/10, 1/100, 1/1000 and 1/10,000 before the spike-in material (5 μl of Hirt lysate diluted 1:1000 from one 15 cm plate of *Drosophila* S2 cells) was added. Subsequent steps were performed as previously described (20).

### Statistical and data analyses

*Adaptor trimming, removal of PCR duplicates, alignment:* XR-seq reads were trimmed to remove adaptor sequences by Cutadapt (46), and then duplicated reads were removed by fastx_toolkit/0.0.14 (hannonlab.cshl.edu/fastx_toolkit/index.html). Trimmed reads were aligned to the human (hg38_UCSC), *Drosophila* (dm6_UCSC), or *C. elegans* (ce11 ENSEMBL (WBcel235, Gen Bank assembly accession: GCA_000002985.3) genomes using Bowtie2 with arguments -f -very-sensitive (47).

#### Nucleotide and dinucleotide distribution

Aligned reads of wild-type CPD XR-seq were filtered to 19–30nts in length and nucleotide composition of each position were plotted for 12 different lengths of excised oligomers by R/4.1.3 with a custom script. For downstream analysis, we selected a range with the best pyrimidine enrichment at the expected damage positions (Supplementary Figure S4). This range is 24–30nt for human NHF1, 20–28nt for *C. elegans* and 25–30nt for *D. melanogaster*. To facilitate comparison across species, all the reads of these indicated lengths were trimmed to 20nt from the 5′ end. Replicates for each sample were combined, mitochondrial reads were extracted to serve as an internal control for assay specificity. Dinucleotide distribution of both genomic and mitochondrial reads was plotted with R using a custom script.

#### Plotting strand-base average repair profiles of the genes

For NHF1, genes longer than 5 kb and at least 5 kb away from the nearest gene were chosen. As per these criteria, the total number of selected genes was 10100. For *C. elegans*, genes longer than 1kbp and at least 500 bp away from the nearest gene were picked. Based on these criteria, the total number of selected genes was 7061. For *D. melanogaster*, genes longer than 1 kb and at least 100 bp away from the nearest gene were selected, resulting in a total of 6218 genes. Each gene was evenly divided into 100 bins from the Transcription Start Site (TSS) to the Transcription End Site (TES) and 25 bins (2 kbp) upstream of TSS, 25 bins (2 kb) downstream of TES. XR-seq reads with the defined read lengths were further filtered to minimize the background by selecting the reads with dipyrimidine between the 4th and 10th position from the 3′ end. Bed files of the reads with these criteria were intersected to the 150 bin-divided-gene list by Bedtools intersect with the following commands -d -wa -F 0.5 -S or -s for TS and NTS, respectively (48). The top 5000 genes were selected based on the wild-type TS/NTS read number ratio for each organism, and these 5000-gene lists were used to create plots. For each individual bin, an average value for each of the selected genes was obtained for both strands. The y axis average reads per kbp per million total reads (RPKM) for each bin was plotted with R.

#### Quantitative spike-in qXR-Seq analysis

In both NHF1 and *C. elegans* qXR-Seq experiments, the spike-in material was obtained from *D. melanogaster*. For the *D. melanogaster* experiments, *C. elegans* was the spike-in. For each sample, raw reads were aligned to the two genomes as previously described. The read numbers, filtered based on length and dipyrimidine content, for both spike-in and the main sample, were used to quantify the relative amount of repair activity.

#### Statistical analysis and figure preparation

Descriptive statistics of the survival and slot blot data were performed and plotted using GraphPad Prism 9, Version 9.4.1. BioRender.com was used to create the figures.

### Data availability/sequence data resources

The raw data have been deposited in the Sequence Read Archive (SRA) of the National Center for Biotechnology Information (NCBI) under accession number PRJNA1013120. A complete list and description of the samples is provided in Supplemental Table S2.

## Results

### Phylogenetic analysis of XPA

It has been reported that the genomes of some green plants do not have a homolog of the *XPA* gene (16), and we wished to examine the extent of *XPA* gene loss in all eukaryotes. We performed an exhaustive phylogenetic analysis to infer the evolutionary history of *XPA* across all eukaryotic species with sequenced genomes and were able to assign multiple *XPA* gene deletion events (Figure 1). The outside circles of this phylogenetic tree indicate the presence of the excision repair genes *XPA* (light blue inner) and *XPC* (mauve outer) in the indicated clades of life. We included the *XPC* gene as a control for genome quality, since species lacking the genes (white) of both excision repair factors are likely to have incomplete genomes.

To identify the XPA-lacking species, we first determined the XPA clade in the tree. We performed an extensive analysis on the tree and MSA to eliminate the paralogous sequences. Our add_lineage.py script adds the most common lowest taxonomic level of clades to each node and the label_duplication_node.py script, counts common species between clades and annotates the nodes as duplication nodes if there is more than one common species. We used this information to pinpoint duplication and deletion events. We also investigated the domain architectures of the BLAST hits and removed the remote homologs with different domain architectures, suggesting a different protein function. According to InterPro data (49), XPA characteristically has a conserved XPA_N and an XPA_C domains. In addition, Sugitani et al. has shown that the positions between amino acids 219–239 are also important in the function of human XPA (50). With all of this, we observed that the cysteine residues at 105–108-126–128-261–264 positions are highly conserved in our alignment (Supplementary Figure S1). The conserved residues guided us to eliminate the remote homologs that are unlikely to perform the XPA function. According to our *hmmscan* results, the clade of our outgroup protein had a common domain (cation efflux) that XPA doesn’t have, and this clade was clustered with another one that several of its proteins also had some other domains (F-box, F-box-like). Even though the F-box clade had some XPA_N and XPA_C hits in *hmmcan* result; it was aligned poorly, particularly in the well-conserved regions; it lacked conserved cysteine residues and clustered with cation efflux clade. In addition, these two outgroup clades had a set of species similar to the XPA clade. With these lines of evidence, we determined those clades as our outgroup and rerooted and pruned the tree accordingly. After this step, we reconstructed a refined MSA and ran PSI-BLAST with the curated MSA as a query. We obtained an additional 164 sequences that were not in our first BLAST output, added them to MSA and built the final XPA tree. We manually curated the final MSA and removed a clade which was poorly aligned and did not meet the defined XPA requirements. With the exclusion of non-XPA proteins, from 1725 species we accepted 1839 proteins as homologs of XPA. Because of the missing species in the species tree (41 species), we were able to show 1796 proteins from 1684 species in our analysis. Lastly, XPC (found in 1994 of 2132 species) was chosen as a control because of its crucial role in global genome repair (4). The full list of the phyletic distribution of the proteins can be found in Supplemental Table S1.

Our results indicate that deletion events during evolution have resulted in the lack of a homolog of *XPA* in most species in the Viridiplantae green plant kingdom, green algae, including *Chlamydomonas* and red algae. There are two exceptions in green plants, *Rhodamnia argentea* and *Carpinus fangiana*, which have an XPA homolog, but these proteins are distinctly located in the metazoan and fungal XPA clades, respectively, and are undoubtedly XPA homologs as their XPA_C and XPA_N domains are well-preserved, and they have the cysteine residues that are conserved across the XPA family. Phyletic placement of these two plant *XPA* genes suggests horizontal gene transfer or simple contamination during sequencing. Like many eukaryotic species, plants also have an *XPC* homolog. Another XPA-lacking clade is Discoba. These excavatum species, including *Trypanosoma*, also have an XPC homolog. Chromalveolata is a eukaryotic group distinctly placed from plants, fungi, and animals in the eukaryotic lineage. Some species in this clade have an XPA homolog. The XPA sequences in the SAR group have some degree of divergence from the rest of the XPA sequences. Yet, they carry the most conserved cysteine residues, a signature of the XPA protein. The presence of XPA in the SAR clade and other eukaryotic supergroups such as Amorphea and Haptista suggests that XPA was present in the common ancestor of eukaryotes.

### Investigation into the requirement of XPA across species with qXR-Seq

**Figure 2.**
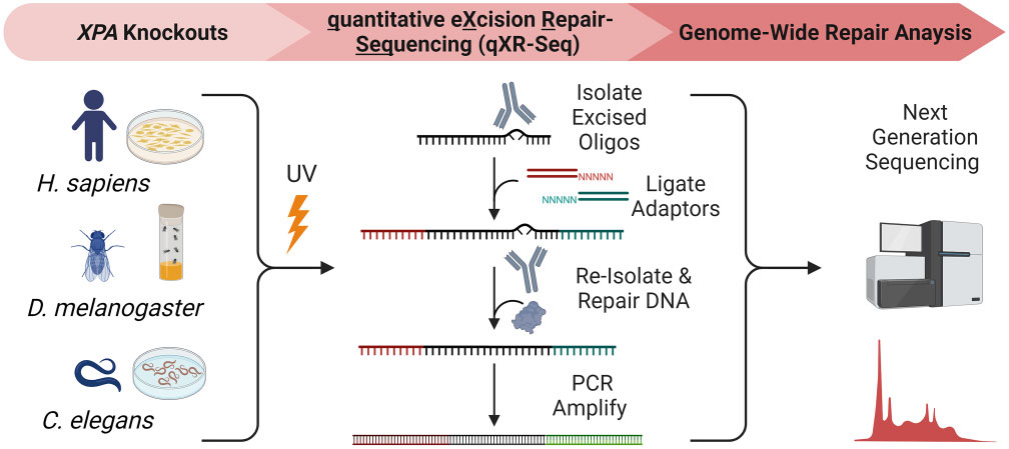
Experimental design for measuring nucleotide excision repair in the absence of XPA in three different organisms. CRISPR technology was used to delete XPA in a human fibroblast cell line (NHF1) and from *Drosophila melanogaster* for this study, and a well-studied *Caenorhabditis elegans* strain with mutated *xpa* was obtained. The qXR-Seq method involves spiking in a constant amount of excised oligos from a different species in order to determine relative amounts of excised oligos between samples. UV-irradiated fly extract was spiked into both human and worm samples, whereas UV-irradiated worm extract was spiked into fly samples. The spike-in was done before the ‘Isolate Excised Oligos’ step so that oligos from both species can be immunoprecipitated with anti-damage antibodies, ligated to adaptors, re-isolated with anti-damage antibodies, repaired with photolyase, and then PCR amplified with bar-coded adaptors. After next generation sequencing, the unique 20–30 nucleotide-long reads containing a dipyrimidine 4–10 nucleotides from the 3′-end were mapped to the genome.

Since excision repair has been reported to occur in organisms lacking an *XPA* homolog (17–19), we wished to determine whether excision repair could also occur in organisms with *XPA* once the gene is knocked out. Figure 2 shows the experimental scheme designed to investigate excision repair in the absence XPA in human fibroblasts and in two model organisms *Drosophila melanogaster* and *Caenorhabditis elegans*. After UV-irradiation of the cells or whole animals, we used the sensitive high throughput sequencing qXR-Seq method to capture and identify the excised oligomers with next generation sequencing to measure repair throughout the genome in a quantitative manner.

### Excision repair in human XPA^−/−^ cells

In humans, the two mechanistic excision repair pathways, global repair and transcription-coupled repair (TCR), depend on XPA-XPG, with the exception of XPC which is not required for TCR, but it requires additional proteins including CSA and CSB (51). TCR, which is defined as greater repair of the transcribed strand (TS) than the non-transcribed strand (NTS) of genes actively transcribed by RNA Polymerase II (RNAPII) (52), acts solely on the transcribed strand independently of the presence or absence of the XPC protein (53,54). TCR can most clearly be observed in XPC-mutant cells (42) because global repair, which does not exhibit strand preference, is the dominant pathway in humans, especially for (6–4)PPs, and thus TCR is somewhat masked. In a previous study we constructed XPC^−/−^/CSB^−/−^ human cell lines and unexpectedly found that these double-mutant cells carried out TCR comparable to XPC mutant cells in terms of the TS/NTS repair ratio (20). Quantitative spike-in qXR-Seq experiments allowed us to determine that excision repair in XPC^−/−^/CSB^−/−^ cells was extremely low, approximately 300-fold less efficient than in wildtype cells. In the current study we wished to conduct qXR-Seq analysis with human cells lacking XPA to determine whether any repair activity could be detected. Thus, we knocked out XPA (Supplementary Figure S2A) in both the normal human fibroblast cell line, NHF1, and the previously generated NHF1/XPC^−/−^/CSB^−/−^ double-mutant cell line (20) to obtain NHF1/XPA^−/−^ and NHF1/XPC^−/−^/CSB^−/−^/XPA^−/−^ cells.

**Figure 3.**
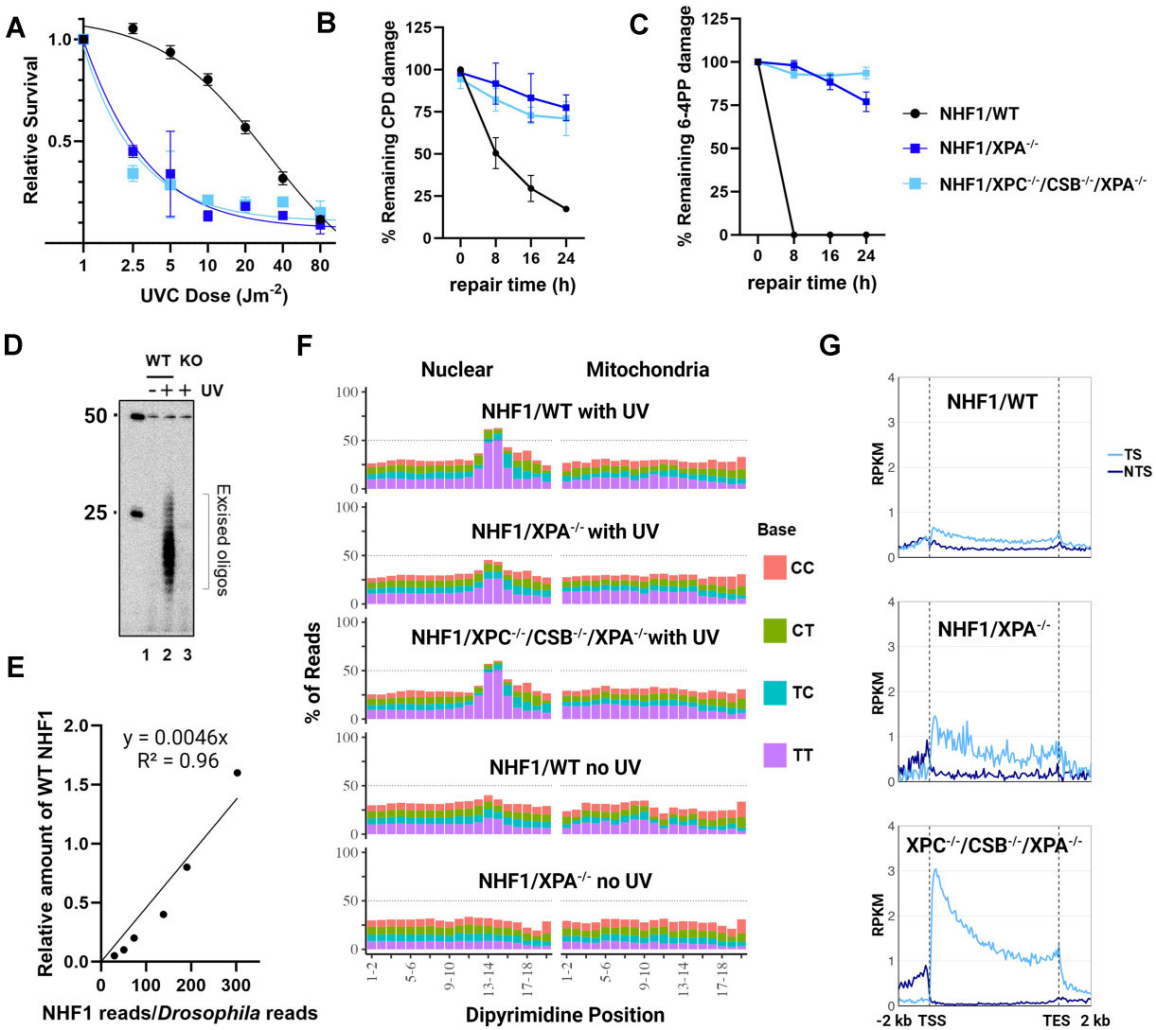
Human NHF1/XPA^−/−^ and NHF1/XPC^−/−^/CSB^−/−^/XPA^−/−^ cells exhibit extreme UV sensitivity and nearly undetectable repair by low resolution assays yet demonstrate evidence of excision repair by qXR-Seq analysis. (**A**) The three NHF1 cell lines were analyzed for survival two days after the indicated doses of UVC. Shown are the mean from three biological replicates with error bars denoting standard error of the mean (SEM). Slot blot analysis showing the (**B**) CPD and (**C**) 6–4PP repair rates of the three NHF1 cell lines treated with 5 J/m^2^ UVC. Damaged-DNA signals were normalized to time = 0 and plotted as a function of time. Results shown are the mean from three biological replicates with error bars denoting SEM. (**D**) The *in vivo* excision assay was used to compare the amount of 6–4PP-containing excised oligos in extracts from the NHF1 wildtype (WT) and NHF1/ XPA^−/−^ (KO) cell lines. An equal number of cells were either unirradiated (lane 1) or irradiated with 20 J/m^2^ UVC (lanes 2 and 3) and incubated 2h at 37°C to allow for repair. Cells were lysed by the Hirt procedure and low molecular weight DNA in the supernatant was immunoprecipitated with anti-6–4PP antibodies. The recovered oligos were mixed with a 50-mer internal control oligo, 5′-end labeled, and separated on a DNA sequencing gel along with the indicated size markers. (**E**) The qXR-seq method was used to map the genomic location of excision products in a quantitative manner. Excised oligos were isolated from human cells lysed with the Hirt method, mixed with excised oligos from Hirt-lysed UV-irradiated *Drosophila* cells, then purified with anti-(6–4)PP specific antibodies, ligated to adapters, and again purified with anti-(6–4)PP antibodies. The damage was reversed with (6–4)PP photolyase and PCR was performed to generate libraries for high throughput sequencing. The sequencing reads were uniquely mapped to either the human or fly genomes, and the graph shows the human:fly ratio relative to the amount of WT NHF1. (**F**) Analysis of the frequency of the possible dipyrimidines along CPD qXR-Seq reads of 24–30nt length (trimmed to 20nt from the 5′ end) from the indicated NHF1 cell lines mapped to either nuclear genome DNA (left) or mitochondrial DNA (right). Mitochondrial DNA analysis and unirradiated cells were included to control for specificity. (**G**) Analysis of transcription-coupled repair (TCR) in the indicated cell lines. CPD qXR-Seq data is plotted as average repair reads (y-axis) along the length of a ‘unit gene’ (x-axis) (5000 genes were selected and divided into 100 bins as described in Materials and methods. The median length for these 5000 genes is 26 196 bp). TCR can clearly be observed in NHF1/XPA^−/−^ cells (middle) even though the plots are not as smooth due to low read numbers. RPKM, reads per kilobase per million mapped reads; TSS, transcription start site; TES, transcription end site; TS, transcribed strand; NTS, nontranscribed strand.

First, we analyzed the survival of the NHF1/XPA^−/−^ and NHF1/XPC^−/−^/CSB^−/−^/XPA^−/−^ cell lines after exposure to different UV doses and found that both mutant lines are extremely sensitive to UV (Figure 3A), as has been previously observed with other mammalian XPA^−/−^ cell lines (21,22,55,56). To compare the rate of UV-adduct removal in the three NHF1 cell lines, the slot blot method with damage-specific antibodies was used to measure the dynamic loss of total genomic DNA damage. After irradiating the cells with 5 J/m^2^ of UVC, we observed that about half of the CPDs (Figure 3B) and all of the (6–4)PPs (Figure 3C) are removed within 8h in wildtype (WT) NHF1, but both NHF1/XPA^−/−^ and NHF1/XPC^−/−^/CSB^−/−^/XPA^−/−^ cells required longer than 24h for half of either UV-photoproduct to be repaired. Thus, with the caveat that measurements at late timepoints are confounded by dilution because of cell division or cell death, we conclude that repair of UV-induced DNA damage is not detectable in XPA^−/−^ cells with this assay. We used another assay called ‘the *in vivo* excision assay’ to directly compare nucleotide excision repair between the WT and XPA^−/−^ cell lines, and as can be seen from the radiolabeled excised oligos in Figure 3D, the levels of excised oligos captured from the XPA^−/−^ knockout (KO) cells (lane 3) is close to the background signal seen in the unirradiated WT cells (lane 1), again indicating that there is little to no excision repair in XPA^−/−^ cells.

Next, we applied our sensitive qXR-Seq assay to the WT and XPA-mutant human cell lines in the presence and absence of UV. All samples had an equal amount of UV-irradiated *Drosophila* extract spiked in so that the excised oligos could be quantitated relative to the linear range of UV-irradiated WT samples (Figure 3E). The equation derived from the WT dilution was used to determine the percentage of excised oligos in the different samples relative to UV-irradiated WT (0.001% in WT no UV; 0.0003% in XPA^−/−^ no UV; 0.001% in XPA^−/−^ with UV; 0.0007% in XPC^−/−^/CSB^−/−^/XPA^−/−^ with UV). Surprisingly, although the relative amount of excision repair in XPA^−/−^ is equivalent to unirradiated wildtype cells, it is 3.6-fold higher than the negative control, unirradiated NHF1/XPA^−/−^ cells. The triple knockout NHF1/XPC^−/−^/CSB^−/−^/XPA^−/−^ cells have even less excised oligos than found in unirradiated WT cells but is approximately 2.4-fold over background (NHF1/XPA^−/−^ no UV).

Analysis of the sequence composition of excised oligos (Figure 3F) indicates that even though repair in the UV-irradiated XPA^−/−^ cell lines is near background, the captured oligos exhibit properties consistent with removal through the excision repair mechanism as currently understood. The sequencing reads were mapped to either nuclear (left) or mitochondrial (right) human genomes, with the latter serving as a negative control. The mitochondrial DNA fragments do not exhibit the base distribution seen in the excised oligos that map to the nuclear genome from all three UV-irradiated cell lines, i.e. a dipyrimidine peak 5–6 nt from the 3′ termini. There does appear to be a slight peak of dipyrimidines in the nuclear-mapped oligos from WT cells which were not UV-irradiated which might either result from excision repair of DNA damage induced by background UV exposure received from light in the laboratory or from indexing barcode misassignment which we have observed in samples with very low read numbers such as these. We did not observe a dipyrimidine peak in the unirradiated WT control in our previous study (20), which is likely due to the differences in the procedures. The original XR-Seq procedure (57) was used in the first study and qXR-Seq was used here, with different cell lysis conditions and antibodies (anti-TFIIH antibodies were used for the first immunoprecipitation in XR-Seq and anti-(6–4)PP antibodies were used here). The dipyrimidine peak is not seen in unirradiated XPA^−/−^ mutant cells which would have received the same background UV exposure. This is likely because the level of repair in these cells is too low for the oligos to be detectable over background noise.

An additional property of nucleotide excision repair is preferential repair of damage in the transcribed strand of genes because of the TCR sub-pathway. Figure 3G shows the analysis of genome-wide repair of UV-induced CPDs in the TS and NTS of wild-type (NHF1/WT) and NHF1/XPA^−/−^ and NHF1/XPC^−/−^/CSB^−/−^/XPA^−/−^ cells. As can be seen from the figure, and in agreement with previous results (20,42), WT NHF1 cells (top) exhibit only a small amount of TCR due to predominant global repair. Nevertheless, TCR can clearly be observed in NHF1/XPA^−/−^ cells (middle), and deletion of XPA from NHF1/XPC^−/−^/CSB^−/−^ cells does not alter the pronounced TCR phenotype (bottom) that we had previously observed in the NHF1/XPC^−/−^/CSB^−/−^ parental cell line (20). This pronounced TCR accentuates the skewed repair pattern observed in the TS from the 5′ end towards the 3′ end of the genes, which is largely masked by global repair in WT NHF1 cells. The skewed pattern gradually diminishes as the repair process proceeds over time (30) which can be explained by the TCR model proposed by Chiou *et al.* (58). Taken together, these results indicate that a low level of both global and transcription-coupled repair occurs in the absence of XPA in human cells.

### Excision repair in xpa-1 *C. elegans*

**Figure 4.**
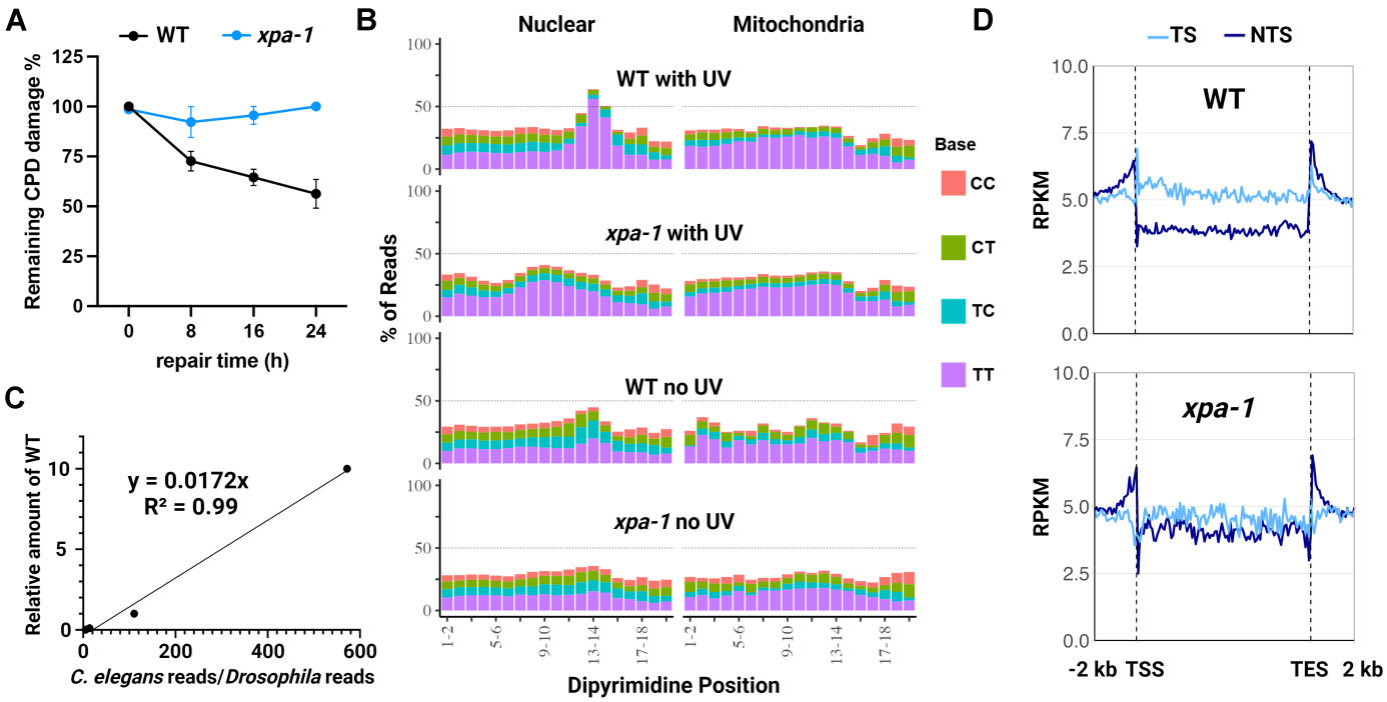
Evidence of excision repair in *xpa-1 C. elegans* revealed by CPD qXR-Seq. **(A)** CPD repair rates of the wildtype and *xpa-1* worms treated with 2000 J/m^2^ UVB. Damaged-DNA signals were normalized to time = 0 and plotted as a function of time. Results shown are the mean from three biological replicates with error bars denoting SEM. (**B**) Analysis of the frequency of the possible dipyrimidines along reads of 20–28 nt length (trimmed to 20nt from the 5′ end) from the indicated worm strains and UV-conditions mapped to either nuclear genome DNA (left) or mitochondrial DNA (right). (**C**) The spike-in analysis of the worm:fly read ratio from the UV-irradiated WT dilution was used to determine the percentage of excised oligos in the different samples relative to WT. (**D**) Analysis of transcription-coupled repair (TCR) in the wildtype (top) and *xpa-1* mutant (bottom) worm strains. CPD qXR-Seq data is plotted as average repair reads (y-axis) along the length of a ‘unit gene’ (x-axis) as described in Figure 3G (The median length for the selected genes is 2245 bp). WT, wildtype; RPKM, reads per kilobase per million mapped reads; TSS, transcription start site; TES, transcription end site; TS, transcribed strand; NTS, nontranscribed strand.

Worms have homologs of all of the human excision repair factors CSA, CSB and XPA-XPG, except for XPE (DDB2), and exhibit both global repair and TCR (10). To determine whether worms excise UV-induced DNA damage in a manner similar to humans, and moreover, whether XPA is required, we obtained wildtype and *xpa-1 Caenorhabditis elegans* strains. First, we performed slot blot analysis with damage-specific antibodies to compare the rate of UVB-induced CPD adduct removal in the two strains (Figure 4A). After irradiating *C.elegans* in the L1 larvae stage with 2000 J/m^2^ UVB, about half of the CPDs are removed within 24h in wildtype (WT) worms, but we do not detect any repair in the *xpa-1* strain with this assay. Next we performed CPD qXR-Seq and found the sequence composition of the excised oligos (Figure 4B) to be very similar in the WT worms to what we had observed in WT human cells, with a clear dipyrimidine peak 5–6 nt from the 3′ termini of reads mapping to the nuclear genome (left) but not the mitochondrial genome (right) of the UV-irradiated worms and a very minor dipyrimidine peak in unirradiated worms. The dipyrimidine enrichment in the UV-irradiated *xpa-1* worms was not as evident as it had been in human XPA^−/−^ cells, but when the worm:fly spike-in read ratio was analyzed relative to the wildtype curve (Figure 4C), there were 10-fold more excised oligos in the UV-irradiated *xpa-1* worms (0.003% relative to UV-irradiated WT) than the unirradiated *xpa-1* worms (0.0003%), which was a larger difference than the 3.6-fold we had measured in humans. Also, like what we observed in the human cell lines, the UV-irradiated *xpa-1* and unirradiated WT worms had levels of excised oligos similar to each other (0.003%). We suspect that the dipyrimidine enrichment in the worm experiments may be partially obscured by higher background in this organism possibly due to a high amount of cell death in the *xpa-1* mutants or due to the much larger number of mutant worms used in the experiment relative to WT worms. Figure 4D shows analysis of genome-wide repair of UV-induced CPDs in the TS and NTS in both strains. The WT worms (top) clearly showed preferential repair of the TS strand indicating robust TCR, and though greatly reduced, the repair strand difference is still evident in the *xpa-1* mutant (bottom) which is further evidence of a low level of repair in the XPA-deficient worms.

### Excision repair in XPA^KO^ drosophila

**Figure 5.**
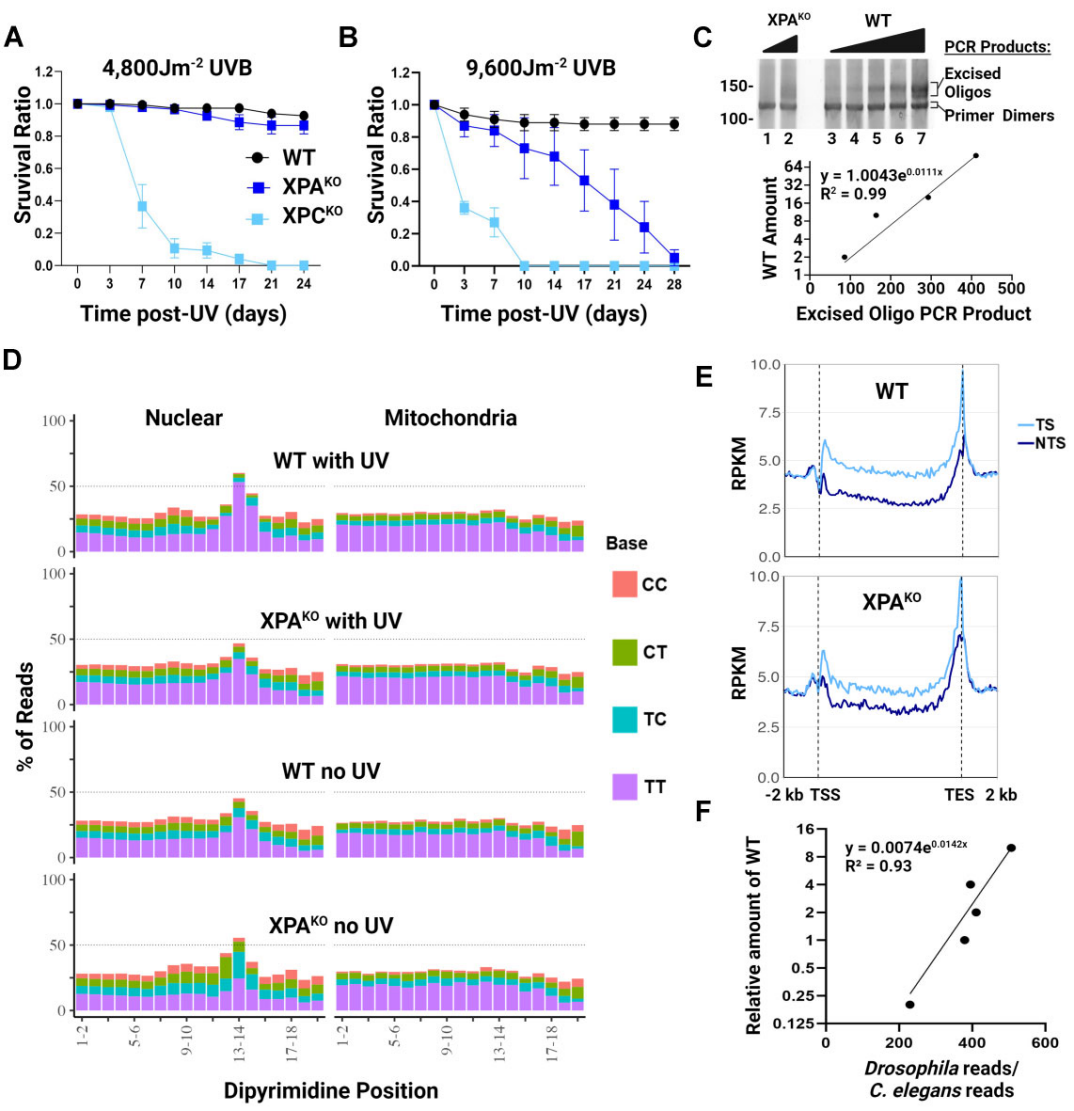
Clear evidence of excision repair without XPA in *Drosophila* revealed by CPD qXR-Seq. (**A, B**) The three indicated fly strains were analyzed for survival for up to four weeks after the indicated doses of UVB. Shown are the mean from three biological replicates with error bars denoting SEM. (**C**) Analysis of dsDNA libraries of the excised CPD-containing oligos by polyacrylamide gel electrophoresis. Ligation products were PCR-amplified with fifteen cycles, and the PCR product descriptions are indicated on the right, sizes (base pairs) on the left, and quantitation of WT is shown below. (**D**) Analysis of the frequency of the possible dipyrimidines along reads of 25–30 nt length (trimmed to 20nt from the 5′ end) from the indicated fly strains and UV conditions mapped to either nuclear genome DNA (left) or mitochondrial DNA (right). (**E**) Analysis of transcription-coupled repair in the WT (top) and XPA^KO^ (bottom) fly strains. CPD qXR-Seq data is plotted as average repair reads (y-axis) along the length of a ‘unit gene’ (x-axis) as described in Figure 3G (The median length for the selected genes is 3024 bp). (**F**) The spike-in analysis of the fly:worm read ratio from the UV-irradiated WT dilution was used to determine the percentage of excised oligos in the different samples relative to WT. WT, wildtype; KO, knockout; J, Joules; RPKM, reads per kilobase per million mapped reads; TSS, transcription start site; TES, transcription end site; TS, transcribed strand; NTS, nontranscribed strand.

**Figure 6.**
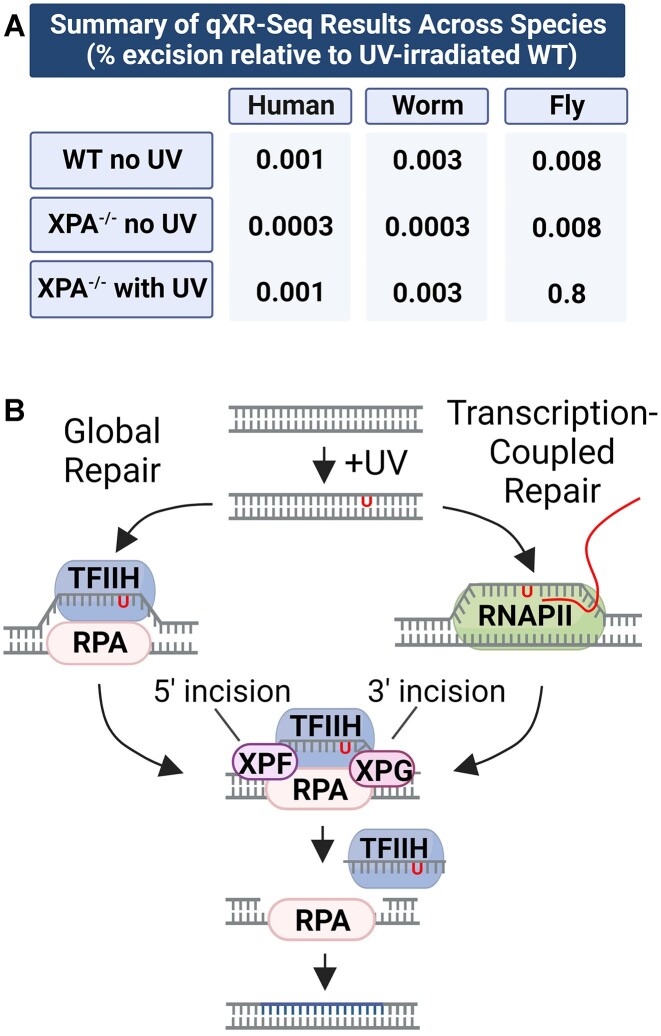
(**A**) Summary of cross-species relative excision repair as determined by qXR-seq. (**B**) Model for global and transcription-coupled repair (TCR) with a ‘minimal essential set’ of excision repair factors proteins. During global repair (left) the dual incision complex is assembled at the damage site by binding of RPA and TFIIH to the damage and then subsequent recruitment of XPF and XPG nucleases. During TCR (right), the ‘transcription bubble’ replaces the damage recognition function and enables the assembly of the four excision factors (RPA, TFIIH, XPG, and XPF). Thus, excision repair can occur in the absence of XPA, XPC, and CSB, but is much less efficient.

Flies also have homologs of the excision repair factors XPA-XPG, but curiously lack homologs of the TCR factors CSA, CSB and UVSSA (11), yet still somehow manage to perform robust TCR (29,30), which unlike in humans is XPC-dependent (29). To determine whether flies can excise UV-induced DNA damage in the absence of XPA, we generated XPA^KO^ flies (Supplementary Figure S3). First, we conducted a survival assay with a medium dose of UVB-irradiation (4800 J/m^2^) and found that the XPA^KO^ flies were not very UV-sensitive (Figure 5A). We observed very little death of the UV-irradiated XPA^KO^ flies after 10 days, similar to WT, but in contrast to the repair-deficient XPC^KO^ flies, of which 90% had died by 10 days. However, when we doubled the dose to 9600 J/m^2^, the XPA^KO^ flies were more sensitive than WT, but were still much less UV-sensitive than the XPC^KO^ flies (Figure 5B). These results were surprising, but since survival is a very indirect way to determine the functional requirement of XPA in excision repair, we performed CPD qXR-Seq to directly map and quantitate the amount of excised oligos in XPA^KO^ flies. As can be seen in Figure 5C there are detectable amounts of excised oligo PCR product in the XPA^KO^ fly samples (lanes 1 and 2) in the analytic gel of the DNA that is subsequently sequenced, which extrapolated to be approximately 2% relative to WT (lanes 3–7, and quantitation below). We analyzed the CPD-containing excised oligo sequence composition as done for human and worm samples and found a clear dipyrimidine peak 5–6 nt from the 3′ termini of reads mapping to the nuclear genome in both WT and XPA^KO^ flies, regardless of UV-irradiation (Figure 5D). Significantly, we observed TCR in both WT and XPA^KO^ flies (Figure 5E). Although the repair difference between strands is more pronounced in WT, repair of TS in both WT and XPA^KO^ shows the same pattern with the peaks at 5′ and 3′ ends, which have been explained previously (29). Taken together this indicates that XPA^KO^ flies undergo excision repair, and when the fly:worm spike-in read ratio was analyzed relative to wildtype (Figure 5F), there was approximately 0.8%, which was 100-fold more excised oligos in the UV-irradiated XPA^KO^ flies than either the unirradiated WT or unirradiated XPA^KO^ flies, both 0.008% relative to UV-irradiated WT. These results are not specific to UV-induced CPD damage as similar results were also obtained with (6–4)PP qXR-Seq (Supplementary Figure S5). In conclusion, we observed significantly more excised oligos in the XPA^KO^ flies than we observed in human cell lines or worms lacking XPA (Figure 6A). The reason that flies are much less dependent on XPA for survival and excision repair remains to be determined but can be added to the growing list of differences in the mechanism of excision repair between this model organism and humans.

## Discussion

Although nucleotide excision repair appears to be universal in cellular organisms ranging from bacteria to humans, it has only been studied in a few model organisms (17,29,51,59–62). Following the development of *in vitro* assays to study nucleotide excision repair in mammalian cells, cell-free extracts were fractionated to isolate the proteins/factors necessary for carrying out the repair reaction. These efforts were greatly aided by the presence of human and Chinese Hamster Ovary (CHO) mutant cell lines defective in excision repair. These investigations led to the identification of six factors (XPA, RPA, TFIIH, XPC-HR23B, XPG and XPF-ERCC1) as the minimal essential set for carrying out damage excision in the form of 26–27 nt (median)-long oligomers (5–7). Parallel work in yeast revealed that the yeast counterparts of these (Rad14, RPA, TFIIH, Rad4-Rad23, Rad2 and Rad1-Rad10) constituted the minimal essential set for excision by dual incision in eukaryotic cells (8). The only protein with an exclusive function in excision repair, as opposed to the other core factors that participate in other cellular functions, is XPA/Rad14, and thus it has been generally assumed to be an essential factor in nucleotide excision repair (63). However, whole genome sequencing has failed to reveal XPA homologs in many organisms including the plant *Arabidopsis*, yet we have shown that *Arabidopsis* performs excision by dual incision essentially identical to humans (17,18). Thus, we wished to know the distribution of XPA in the phylogenetic tree and to also inquire whether other organisms such as humans, worms, and flies can carry out excision without XPA, albeit at such a low level that was previously undetectable by the early excision assays.

We found that a large number of eukaryotes that have the other ‘core excision repair factors’ lack XPA. Moreover, we find that in *Drosophila*, and to a lesser extent even in humans and *C. elegans*, excision by the diagnostic dual incision generating 26–27-mers does occur in all three species. The level of excision is most prominent in *Drosophila*, but it is still only ∼1% of that found in wildtype flies. Whether this value and the much lower excision in humans and *C. elegans* have any physiological significance is questionable. It is important to note that other modes of nucleotide excision repair-independent UV photoproduct removal have been reported involving Topoisomerase I (64) or APE1 (65), however these do not produce the dipyrimidine-containing 20–30 nucleotide oligonucleotides with the diagnostic 5′ and 3′ incision sites that we are mapping with qXR-Seq and thus are not relevant to our analysis of repair factor requirement for nucleotide excision repair in its commonly accepted form.

We conclude that even though XPA is not essential, it is still a critical component for efficient nucleotide excision repair in humans, worms, and flies. XPA acts cooperatively with RPA to recognize DNA damage (66), interacts with TFIIH (67), and recruits XPF-ERCC1 to the excision repair complex by specifically binding to ERCC1 (68) and its counterpart Rad10 in yeast (69,70). Therefore, the apparent absence of XPA in many organisms, including plants, is rather surprising and remains to be further investigated. Though the amino acid sequence is highly conserved across species with XPA, the protein has been shown to be an intrinsically unstructured (71), and thus it is conceivable that an XPA ortholog not detectable by standard programs participates in plant excision repair or even that a protein unrelated to XPA, but nevertheless possessing similar properties, substitutes for XPA during excision repair. Our findings provide insight into what is the absolutely ‘minimal essential set’ for excision repair and for the assembly of the excision repair complex (Figure 6B). The ‘minimal essential set’ consists of two damage recognition factors, TFIIH and RPA, and two endonucleases, XPF-ERCC1 and XPG. TFIIH unwinds the DNA around the damage and RPA binds the resulting single-stranded DNA, and then both factors play important roles in recruiting the two nucleases. XPG forms protein-protein complexes with both RPA (66) and TFIIH (5), and *in vitro* studies with bubble substrates have demonstrated that RPA promotes recruitment and junction cutting by both XPF-ERCC1 and XPG nucleases (72). It is clear, however, even though the four-factor minimal set is capable of nucleotide excision repair, the six-factor ensemble of repair proteins is needed in many organisms for biologically relevant repair rates.

## Supplementary Material

gkad1104_Supplemental_Files

## Data Availability

The raw data have been deposited in the Sequence Read Archive (SRA) of the National Center for Biotechnology Information (NCBI) under accession number PRJNA1013120.
