# Supplementary material for: Cross-species investigation into the requirement of XPA for nucleotide excision repair
Source: Nucleic Acids Res. 2023 Nov 22;52(2):677–89. doi: 10.1093/nar/gkad1104 (PMC10810185; doi:10.1093/nar/gkad1104)
Supplement: gkad1104_Supplemental_Files [file gkad1104_supplemental_files.zip › 230920 NAR Supplement.pdf]

SUPPLEMENTAL FIGURES

Cross-Species Investigation into the Requirement of XPA for Nucleotide Excision Repair

Cansu Kose[†,1], Xuemei Cao[†,1], Evan B. Dewey[2], Mustafa Malkoç[3], Ogün Adebali[3,4], Jeff Sekelsky[2], Laura A. Lindsey-Boltz[1,*], Aziz Sancar[1,*]

Supplemental Figure S1.

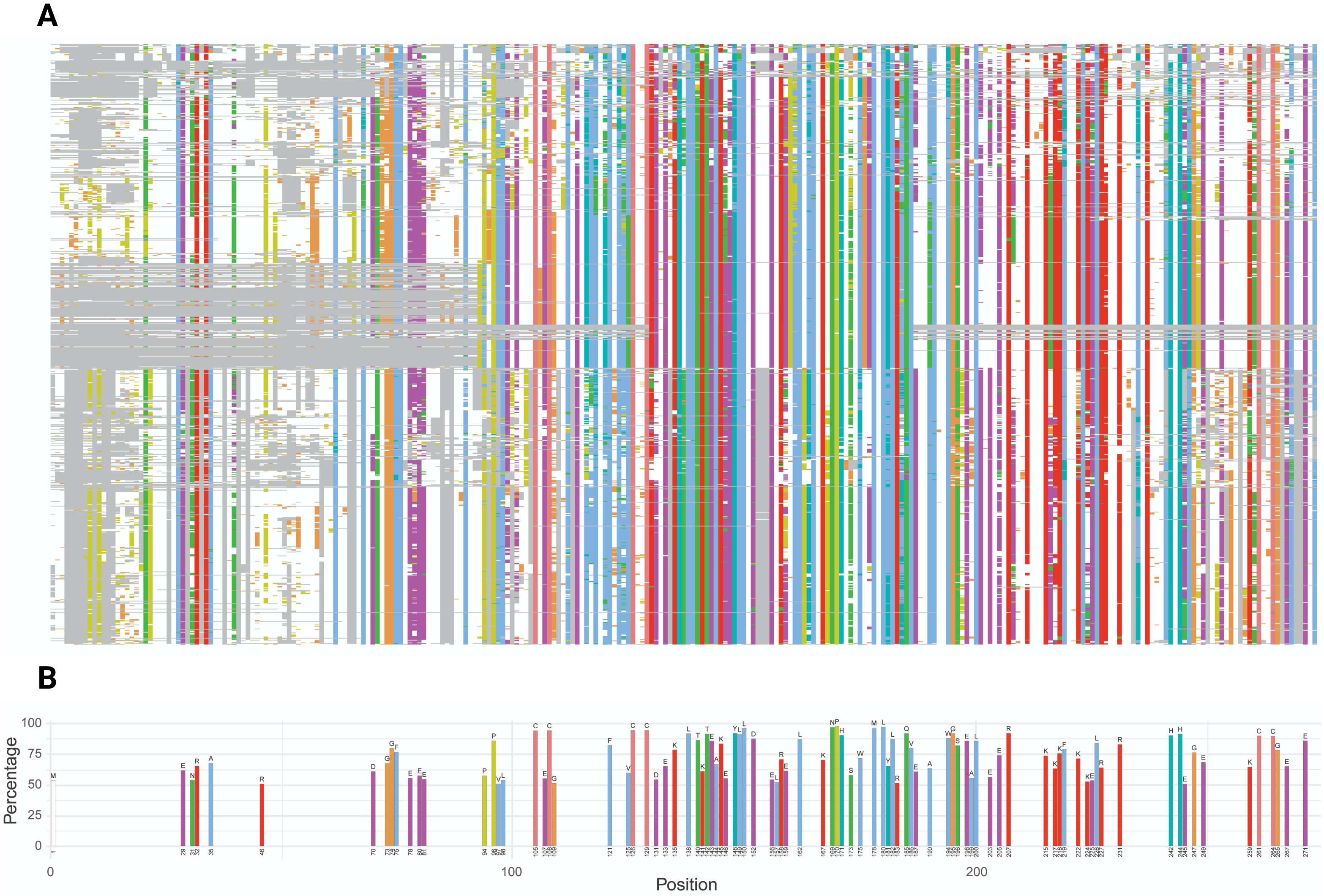

Figure S1. Multiple Sequence Alignment (MSA) of XPA homologs. (A) 1,796 XPA homologs from 1,725 different species from the final XPA tree are shown. Alignment positions with a gap in human XPA are removed from the alignment visualization. Residues conserved with 50% or more are shown in the bar graph **(B)**. For the coloring, "Clustal" option of Jalview (39) was used. The Clustal X color scheme assigns colors to amino acids as follows: blue for hydrophobic, red for positively charged, magenta for negatively charged, green for polar, pink for cysteine, orange for glycine, yellow for proline, cyan for aromatics, white for not conserved, and gray for gaps.

Supplemental Figure S2.

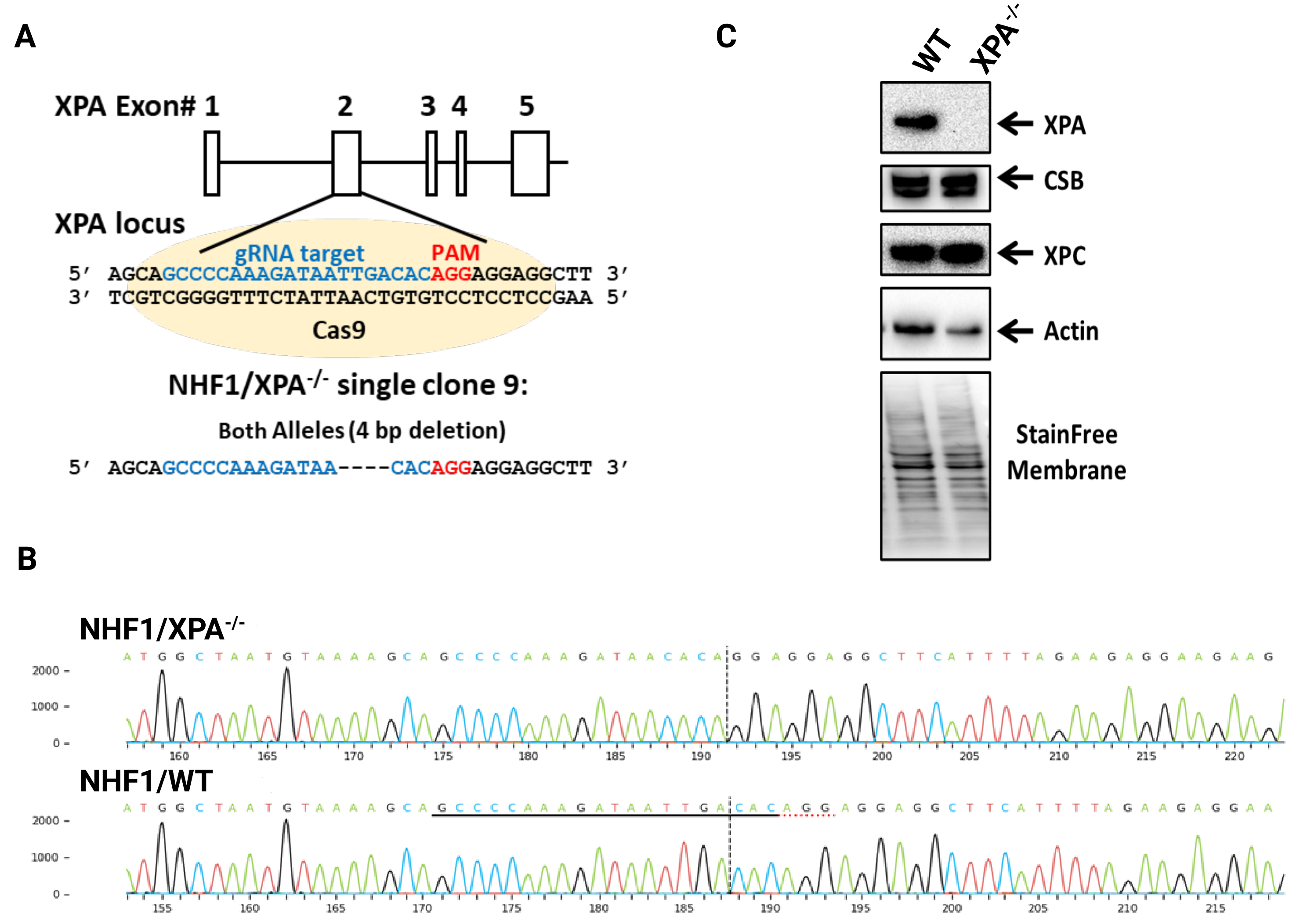

Figure S2. Generation of the NHF1/XPA$^{-/-}$ cell line. (A) CRISPR/Cas9-induced homology directed repair was used to delete XPA from the normal human fibroblast cell line (NHF1/WT). The oligonucleotides sgRNA#2F (5’-CACCGGCCCCAAAGATAATTGACAC-3’) and sgRNA#2R (5’-AAACGTGTCAATTATCTTTGGGGCC-3’) were cloned into the plasmid LentiCRISPRv2 Neo (Addgene 98292), and this plasmid together with the envelope pCMV-VSV-G (Addgene 8454) and packaging psPAX2 (Addgene 12260) plasmids were used to generate the lentivirus targeting the sequence GCCCCAAAGATAATTGACAC (PAM sequence AGG) in exon 2 of XPA. The antibiotic G418 was added to cell cultures diluted so that resistant colonies could be isolated from a single originating cell. **(B)** The *XPA* gene mutation was identified by Sanger sequencing, and **(C)** protein expression by immunoblotting with the following antibodies from Santa Cruz: XPA sc-56813, XPC sc-74410, CSB sc-398022, and Actin sc-1616.

Supplemental Figure S3.

A

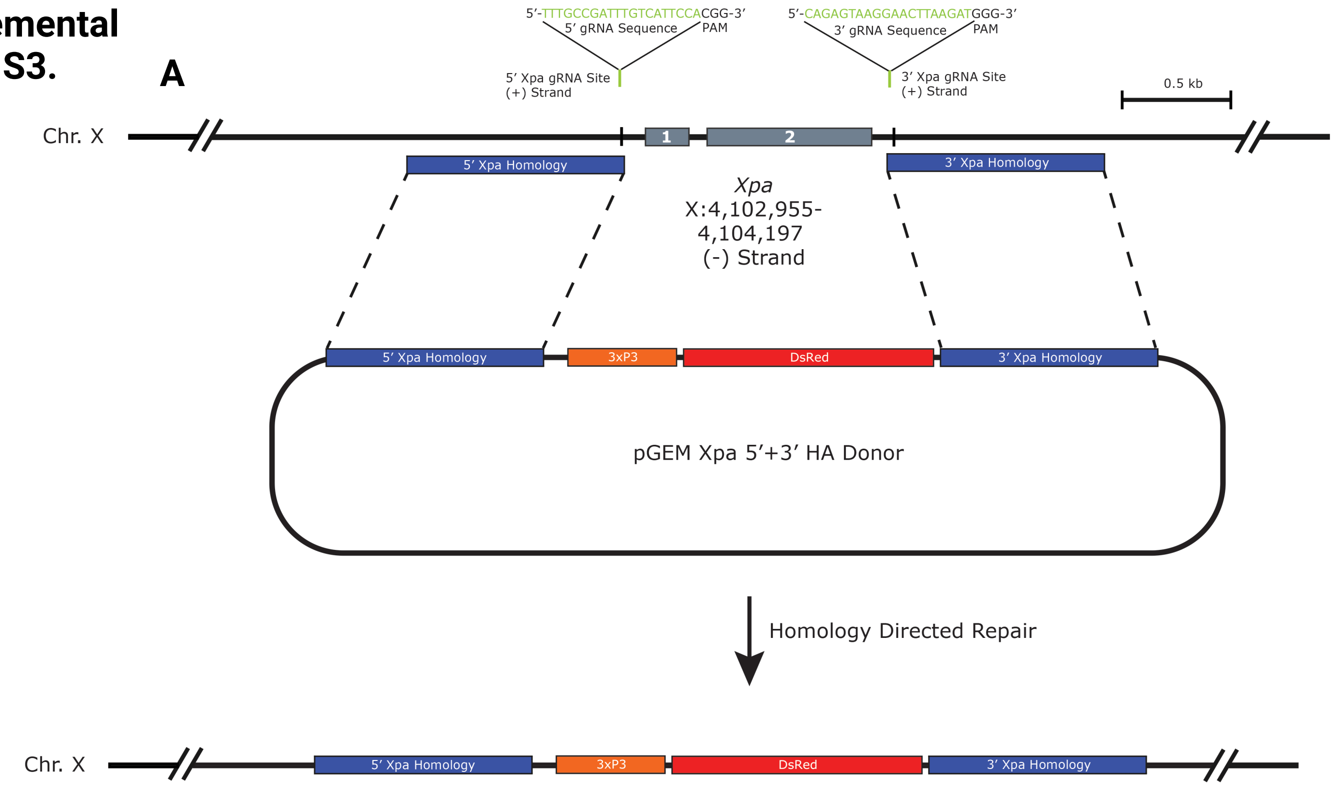

B

5'-AACGAAACGTACCACTTGCTGAACTTGTGGCGGAGCGGGGATGTCTGAAACACGTGCCCGGCGAACAGCAACTCCGGC
TGGTGGCTCTGACGCCCCCTGCCCAGAAACTTGAGCACGTTCTTGGCCGAAACATAGTAGTCGTCGTCCACGAAAAGATAG
AACTCGCTGCGATTGAACTGATCGCTAGCCCAGCGCATACCCAGCATGGTTTTCAGCGTGTTGTTGAAGTAGGCGTCCGTGA
ACTCCGCCTGCAGAATGTCGCCATGCTCGCGGGACTCCCAAGCCACGTCTTTCTCACTATCCTCGGCCGTGCCCAGCAGGAA
CACGCGACGCAGATGGACGTCTGAGAATCGACCCTCGTAGCCCCAAGTCCGGCGAATGGCCTCCCGACGTCGCGAATTGCC
CACCGCGCTCTTTATGAGCATCGTGAGCCGCGCAGGCTGGTCAACCGGCACCTCTGCCGTGAACGAGGGCACTCGCAGGTA
GGCGAACTTGTCCAGTCCAGAGGATGCGGACCCAGATCCAGTGTCATCGTTCAGCGGGTAGTGAAAGTGCCGCTCGAAGT
TCAGCTCGTGCAGGTGGGTCAGCAGGCCGCAATAGTCCACCAGAAGGATGAGGGGCAGCACCAGGAGGCAGCGCAGCA
GCAGTTTGCGGTGTTTACTTTGCATGGCGACCAAAATAAAAACAAATCCGCCAGTGTGACCGTGATGCGGTTAAAATACTAA
ACGCCCATCTACCGTACGAAAAATAAAATAACTAAGACTAAAAGCATTCAAATTGAGTGCTTACAAATCTAAAAAATTGTTTTA
CAGttgaatgagaagtgtgacattgatttatttatatgtaaaagaaaaaaactcttttcacgtatttttacatttccgccaataacagttattttctccgcgtaaaagc
tggctgcactgaacaaacaaatttcTgtggaatgacaaATCGGCAAAgtacggtaccAATTGAGCTCATAACTTCGTATAATGTATGCTATA
CGAAGTTATGTCGACGAATTCGCGGCCGCGAGCTCGCCCGGgatctaattcaattagagactaattcaattagagctaattcaattaggat
ccaagcttatcgatttcgaaccctcgaccgccggagtataaatagaggcgcttcgtctacggagcgacaattcaattcaaacaagcaaagtgaacacgtcgct
aagcgaaagctaagcaaataaacaagcgcagctgaacaagctaaacaatcgggcggccgcactagagccggtcgccaccatgaggtcttccaagaatgtta
tcaaggagttcatgaggtttaaggttcgcatggaaggaacggtcaatgggcacgagtttgaaatagaaggcgaaggagaggggaggccatacgaaggcca
caataccgtaaagcttaaggtaaccaagggggggacctttgccatttgcttgggatattttgtcaccacaatttcagtatggaagcaaggtatatgtcaagcaccc
tgccgacataccagactataaaaagctgtcatttcctgaaggatttaaatgggaaagggtcatgaactttgaagacggtggcgtcgttactgtaacccaggatt
ccagtttgcaggatggctgtttcatctacaaggtcaagttcattggcgtgaactttccttccgatggacctgttatgcaaaagaagacaatgggctgggaagccag
cactgagcgtttgtatcctcgtgatggcgtgttgaaaggagagattcataaggctctgaagctgaaagacggtggtcattacctagttgaattcaaaagtatttaca
tggcaaagaagcctgtgcagctaccagggtactactatgttgactccaaactggatataacaagccacaacgaagactatacaatcgttgagcagtatgaaaga
accgagggacgccaccatctgttcctttagcggccatcgaattcgagctcgcccactaagcgtcgcgccacttcaacgctcgatgggagcgtcattggtgggcggg
gtaaccgtcgaaatcagtgtttacgcttccaatcgcaacaaaaaattcactgcaacactgaaaagcatacgaaaacgatgaagattgtacgagaaaccataaag
tattttatccacaaagacacgtatagcagaaaagccaagttaactcggcgataagttgtgtacacaagaataaaatcggccagattcagtgttgtcagaaataag
aaaaccccactatgtttttctttgccttttctttctcccagcgatcattcatttcgtggtgaaagaacggggtcattgcacggagtttcgactgcgggaaagcagagct
gccgttcacttcgtctataattagcgctttctattttccccgattcgggccgctgctgcgcttttccgcctgctgtttgtggcaagtgtagcagcaggctgtgcacgca
gtgtggcatgcacttggctttccaccgttggtatcgattctctgggacgatgagtcattcctttcggggccacagcataatcgttgccagctcaccgaaatggtga
cttcatttcttaactgccgtcaagcatgcgattgtacatacatacatatttatatatgtacatatttatgtgactatggtaggtcgatataatagcaatcaacgcaa
gcaaatgtgtcagtcctgcttacaggaacgattctatttagtaattttcgttgtataaagtaattatgtatgtatgtaagccccataaatctgaaacaattaggca
aaaccatgcgaagctCTCTGGCGCCTAACGCATCTTAAGTTCCTTACTCTGTAAACTCATGTTACATTAAAAACATAGTTTAGTGA
ATTCTAAGTGAATTATGTAATTGAACTAAACACAAATGCGCATGAATTAAGTGCTAAAATGTGATTGGAATTTGGTTTAAA
ATACAAGATTCTGGTGCATTTCAACACTTCTCCTAGATAACGTCGCGTTTTATATGGCTTCACATTGCGTGGAATTATTTATTC
TGCCATCTGGCGAACATCTCAAGACGGCGCTTGGCCAAGTTGAGATCGCTACCGACGAACATGTACTCTAGACGATCGTATA
ACGACCGCAGACGGCGATCCACATGGAAACGCATCTAATTGACCAACTGCTTAAGCAGTTTAAACGTGTACTCTTTGTTGGG
TATGTTCCATATAAACTTTTTTTCAGCTGGGATCTGATTCAATCCATCAATTGTACGCCCCTCACCAGTGGGATCGTCCGTAAA
GCGCATGTTATCCATGTTGGACACACATAAGCCGGGATATTATGATGCTTTACTGGAATTTTAAGCCACCTATTTTAAAACTTC
TAAACGTGTGAACAGTAAGGTTCTGCTGTTCGAATGCCTGGAACTTCTGACTGCCTTctggaagactaattcaacagctactaaaggct
attgtgatttccttttctcaacctacgcggttgtaagggcgccagcacccgcaattgtatctggaagatagttgctagcaacactgattgtaacatagcgatatgtcc
gggtgctccggaaaaatcggtggtgaaatcccgggttggaggcgttccgcgagggtatataaggcggtaccttggctacaggccattaagtttagaccatttttta
cattttcatgatggtttcaaaaatgcgaaaatttggcaaaaaatttagaatttggcagtaaagtttagttataagcaagaagtaaagttacagttataattaataatt
aaacaacaaattgaacTGGGAATGCAAAGTTTTATTTGAAATTTGTTACAATCATAATGCACCGGGAGCTGATGTG-3'

Supplemental Figure S3.

C

```
Query  1    CGCGTAAAAGCTGGCTGCACTGAACAAACAAATTTCTGTGGAATGACAAATCGGCAAAG-  59
            ||||||||||||||||||||||||||||||||||| ||||||||||||||||||||||| 
Sbjct  1    CGCGTAAAAGCTGGCTGCACTGAACAAACAAATTTCCGTGGAATGACAAATCGGCAAAGT  60

Query  60   ACGGTACCAATTGAGCTCATAACTTTGTATAATGTATGCTATACGAAGTTATGTCGACGA  119
            ||||||||||||||||||||||||| ||||||||||||||||||||||||||||||||||
Sbjct  61   ACGGTACCAATTGAGCTCATAACTTCGTATAATGTATGCTATACGAAGTTATGTCGACGA  120

Query  120  ATTCGCGGCCGCGAGCTCGCCCGGGGATCTAATTCAATTAGAGACTAATTCAATTAGAGC  179
            ||||||||||||||||||||||||||||||||||||||||||||||||||||||||||||
Sbjct  121  ATTCGCGGCCGCGAGCTCGCCCGGGGATCTAATTCAATTAGAGACTAATTCAATTAGAGC  180

Query  180  TAATTCAATTAGGATCCAAGCTTATCGATTTCGAACCCTCGACCGCCGGAGTATAAATAG  239
            ||||||||||||||||||||||||||||||||||||||||||||||||||||||||||||
Sbjct  181  TAATTCAATTAGGATCCAAGCTTATCGATTTCGAACCCTCGACCGCCGGAGTATAAATAG  240

Query  240  AGGCGCTTCGTCTACGGAGCGACAATTCAATTCAAACAAGCAAAGTGAACACGTCGCTAA  299
            ||||||||||||||||||||||||||||||||||||||||||||||||||||||||||||
Sbjct  241  AGGCGCTTCGTCTACGGAGCGACAATTCAATTCAAACAAGCAAAGTGAACACGTCGCTAA  300

Query  300  GCGAAAGCTAAGCAAATAAACAAGCGCAGCTGAACAAGCTAAACAATCGGGCGGCCGCAC  359
            ||||||||||||||||||||||||||||||||||||||||||||||||||||||||||||
Sbjct  301  GCGAAAGCTAAGCAAATAAACAAGCGCAGCTGAACAAGCTAAACAATCGGGCGGCCGCAC  360

Query  360  TAGAGCCGGTCGCCACCATGAGGTCTTCCAAGAATGTTATCAAGGAGTTCATGAGGTTTA  419
            ||||||||||||||||||||||||||||||||||||||||||||||||||||||||||||
Sbjct  361  TAGAGCCGGTCGCCACCATGAGGTCTTCCAAGAATGTTATCAAGGAGTTCATGAGGTTTA  420

Query  420  AGGTTCGCATGGAAGGAACGGTCAATGGGCACGAGTTTGAAATAGAAGGCGAAGGAGAGG  479
            ||||||||||||||||||||||||||||||||||||||||||||||||||||||||||||
Sbjct  421  AGGTTCGCATGGAAGGAACGGTCAATGGGCACGAGTTTGAAATAGAAGGCGAAGGAGAGG  480

Query  480  GGAGGCCATACGAAGGCCACAATACCGTAAAGCTTAAGGTAACCAAGGGGGGACCTTTGC  539
            ||||||||||||||||||||||||||||||||||||||||||||||||||||||||||||
Sbjct  481  GGAGGCCATACGAAGGCCACAATACCGTAAAGCTTAAGGTAACCAAGGGGGGACCTTTGC  540

Query  540  CATTTGCTTGGGATATTTTGTCACCACAATTTCAGTATGGAAGCAAGGTATATGTCAAGC  599
            ||||||||||||||||||||||||||||||||||||||||||||||||||||||||||||
Sbjct  541  CATTTGCTTGGGATATTTTGTCACCACAATTTCAGTATGGAAGCAAGGTATATGTCAAGC  600

Query  600  ACCCTGCCGACATACCAGACTATAAAAAGCTGTCATTTCCTGAAGGATTTAAATGGGAAA  659
            ||||||||||||||||||||||||||||||||||||||||||||||||||||||||||||
Sbjct  601  ACCCTGCCGACATACCAGACTATAAAAAGCTGTCATTTCCTGAAGGATTTAAATGGGAAA  660

Query  660  GGGTCATGAACTTTGAAGACGGTGGCGTCGTTACTGTAACCCAGGATTCCAGTTTGCAGG  719
            ||||||||||||||||||||||||||||||||||||||||||||||||||||||||||||
Sbjct  661  GGGTCATGAACTTTGAAGACGGTGGCGTCGTTACTGTAACCCAGGATTCCAGTTTGCAGG  720

Query  720  ATGGCTG  726
            |||||||
Sbjct  721  ATGGCTG  727
```

D

```
Query  1    TGCTGTTTGTGGCAAGTGTAGCAGCAGGCTGTGCACGCAGTGTGGCATGCACTTGGCTTT  60
            ||||||||||||||||||||||||||||||||||||||||||||||||||||||||||||
Sbjct  1    TGCTGTTTGTGGCAAGTGTAGCAGCAGGCTGTGCACGCAGTGTGGCATGCACTTGGCTTT  60

Query  61   CCACCGTTGGTATCGATTCTCTGGGACGATGAGTCATTCCTTTCGGGGCCACAGCATAAT  120
            ||||||||||||||||||||||||||||||||||||||||||||||||||||||||||||
Sbjct  61   CCACCGTTGGTATCGATTCTCTGGGACGATGAGTCATTCCTTTCGGGGCCACAGCATAAT  120

Query  121  CGTTGCCAGCTCACCGAAATGGTGACTTCATTTCTTAACTGCCGTCAAGCATGCGATTGT  180
            ||||||||||||||||||||||||||||||||||||||||||||||||||||||||||||
Sbjct  121  CGTTGCCAGCTCACCGAAATGGTGACTTCATTTCTTAACTGCCGTCAAGCATGCGATTGT  180

Query  181  ACATACATACATATTTATATATGTACATATTTATGTGACTATGGTAGGTCGATATAATAG  240
            ||||||||||||||||||||||||||||||||||||||||||||||||||||||||||||
Sbjct  181  ACATACATACATATTTATATATGTACATATTTATGTGACTATGGTAGGTCGATATAATAG  240

Query  241  CAATCAACGCAAGCAAATGTGTCAGTCCTGCTTACAGGAACGATTCTATTTAGTAATTTT  300
            ||||||||||||||||||||||||||||||||||||||||||||||||||||||||||||
Sbjct  241  CAATCAACGCAAGCAAATGTGTCAGTCCTGCTTACAGGAACGATTCTATTTAGTAATTTT  300

Query  301  CGTTGTATAAAGTAATTATGTATGTATGTAAGCCCCATAAATCTGAAACAATTAGGCAAA  360
            ||||||||||||||||||||||||||||||||||||||||||||||||||||||||||||
Sbjct  301  CGTTGTATAAAGTAATTATGTATGTATGTAAGCCCCATAAATCTGAAACAATTAGGCAAA  360

Query  361  ACCATGCGAAGCTCTCTGGCGCCTAACGCATCTTAAGTTCCTTACTCTGTAAACTCATGT  420
            ||||||||||||||||||||||||||| ||||||||||||||||||||||||||||||||
Sbjct  361  ACCATGCGAAGCTCTCTGGCGCCTAACCCATCTTAAGTTCCTTACTCTGTAAACTCATGT  420

Query  421  TACATTAAAAACATAGTTTAGTGAATTCTAAGTGAATTATGTAATTGAACTAAACACAAA  480
            ||||||||||||||||||||||||||||||||||||||||||||||||||||||||||||
Sbjct  421  TACATTAAAAACATAGTTTAGTGAATTCTAAGTGAATTATGTAATTGAACTAAACACAAA  480

Query  481  TGCGCATGAATTAAGTGCTAAAATGTGATTGGAATTTGGTTTAAAATACAAGATTCTGGT  540
            ||||||||||||||||||||||||||||||||||||||||||||||||||||||||||||
Sbjct  481  TGCGCATGAATTAAGTGCTAAAATGTGATTGGAATTTGGTTTAAAATACAAGATTCTGGT  540

Query  541  GCATTTCAACACTTCTCCTAGATAACGTCGCG  572
            ||||||||||||||||||||||||||||||||
Sbjct  541  GCATTTCAACACTTCTCCTAGATAACGTCGCG  572
```

E 5’-cgcgtaaaagctggctgcactgaacaaacaaatttcTgtggaatgacaaATCGGCAAAgtacggtaccAATTGAGCTCATA
ACTTCGTATAATGTATGCTATACGAAGTTATGTCGACGAATTCGCGGCCGCGAGCTCGCCCGGggatctaattca
attagagactaattcaattagagctaattcaattaggatccaagcttatcgatttcgaaccctcgaccgccggagtataaatagaggcgcttc
gtctacggagcgacaattcaattcaaacaagcaaagtgaacacgtcgctaagcgaaagctaagcaaataaacaagcgcagctgaacaa
gctaaacaatcgggcggccgcactagagccggtcgccaccatgaggtcttccaagaatgttatcaaggagttcatgaggtttaaggttcgc
atggaaggaacggtcaatgggcacgagtttgaaatagaaggcgaaggagaggggaggccatacgaaggccacaataccgtaaagctta
aggtaaccaaggggggacctttgccatttgcttgggatattttgtcaccacaatttcagtatggaagcaaggtatatgtcaagcaccctgcc
gacataccagactataaaaagctgtcatttcctgaaggatttaaatgggaaagggtcatgaactttgaagacggtggcgtcgttactgtaac
ccaggattccagtttgcaggatggctg

F tgctgtttgtggcaagtgtagcagcaggctgtgcacgcagtgtggcatgcacttggctttccaccgttggtatcgattctctgggacgatgag
tcattcctttcggggccacagcataatcgttgccagctcaccgaaatggtgacttcatttcttaactgccgtcaagcatgcgattgtacataca
tacatatttatatatgtacatatttatgtgactatggtaggtcgatataatagcaatcaacgcaagcaaatgtgtcagtcctgcttacaggaa
cgattctatttagtaattttcgttgtataaagtaattatgtatgtatgtaagccccataaatctgaaacaattaggcaaaaccatgcgaagctC
TCTGGCGCCTAACGCATCTTAAGTTCCTTACTCTGTAAACTCATGTTACATTAAAAACATAGTTTAGTGAATT
CTAAGTGAATTATGTAATTGAACTAAACACAAATGCGCATGAATTAAGTGCTAAAATGTGATTGGAATTTGG
TTTAAAATACAAGATTCTGGTGCATTTCAACACTTCTCCTAGATAACGTCGCG

Figure S3. Replacement of endogenous *Xpa* gene with dsRed via CRISPR/Cas9-induced Homology Directed Repair.(A) Flies expressing Cas9 in male germline stem cells (under control of the *nanos* promoter) were injected with plasmids containing guide RNAs 5' and 3' to the *Xpa* gene on chromosome X (pCFD4 *Xpa* gRNA; under expression of a U6 promoter) and containing donor (template) DNA with 3XP3 (eye) promoter-driven dsRed flanked by homology to regions immediately 5' and 3' of *Xpa* (pGEM *Xpa* 5' + 3' HA Donor; injections by Genetivision, Houston, TX). In successful replacement, the *Xpa* gene (gray boxes) is excised via Cas9 cutting at the 5' and 3' gRNA sites (green) and replaced with dsRed in male germline stem cells by Homology Directed Repair that uses the 5' and 3' homologies (blue) contained on the donor plasmid as a template. Males with replacement in their germline stem cells then transmit this modified, Δ*Xpa*:dsRed chromosome to their progeny, and successful replacement is indicated phenotypically by expression of dsRed in eyes. dsRed-positive male progeny were then isogenized and used to make a Δ*Xpa*:dsRed stock. gRNA sequences (green sequence above green boxes) were generated using the flyCRISPR design tool (flyCRISPR.org), with protospacer adjacent motif (PAM) sequences in black. This figure was generated using SnapGene® (with modifications and additions), and was based on one from Lamb, et al., 2017 (22). **(B)** pGEM *Xpa* 5'+3' Donor plasmid sequence. Underlined regions indicate Sanger sequence obtained from PCR of genomic DNA of the isogenized male used to make the Δ*Xpa*:dsRed stock, indicating successful deletion of *Xpa* and replacement with dsRed. Blue= *Xpa* 5' and 3' homology, Yellow= 3XP3 Promoter, and Red= dsRed. **(C)** and **(D)** Alignments to the 5' and 3' of the *Xpa* gene locus, respectively, of isogenized male Sanger sequence from PCR of genomic DNA indicating alignment to pGEM *Xpa* 5'+3' Donor plasmid sequence and illustrating genomic replacement of *Xpa* with dsRed. **(E)** and **(F)** Sanger sequences of PCR products 5' and 3' to *Xpa* gene locus, respectively, obtained from the isogenized male used to make the Δ*Xpa*:dsRed stock, with mutated PAM site residues in magenta.

Supplemental Figure S4.

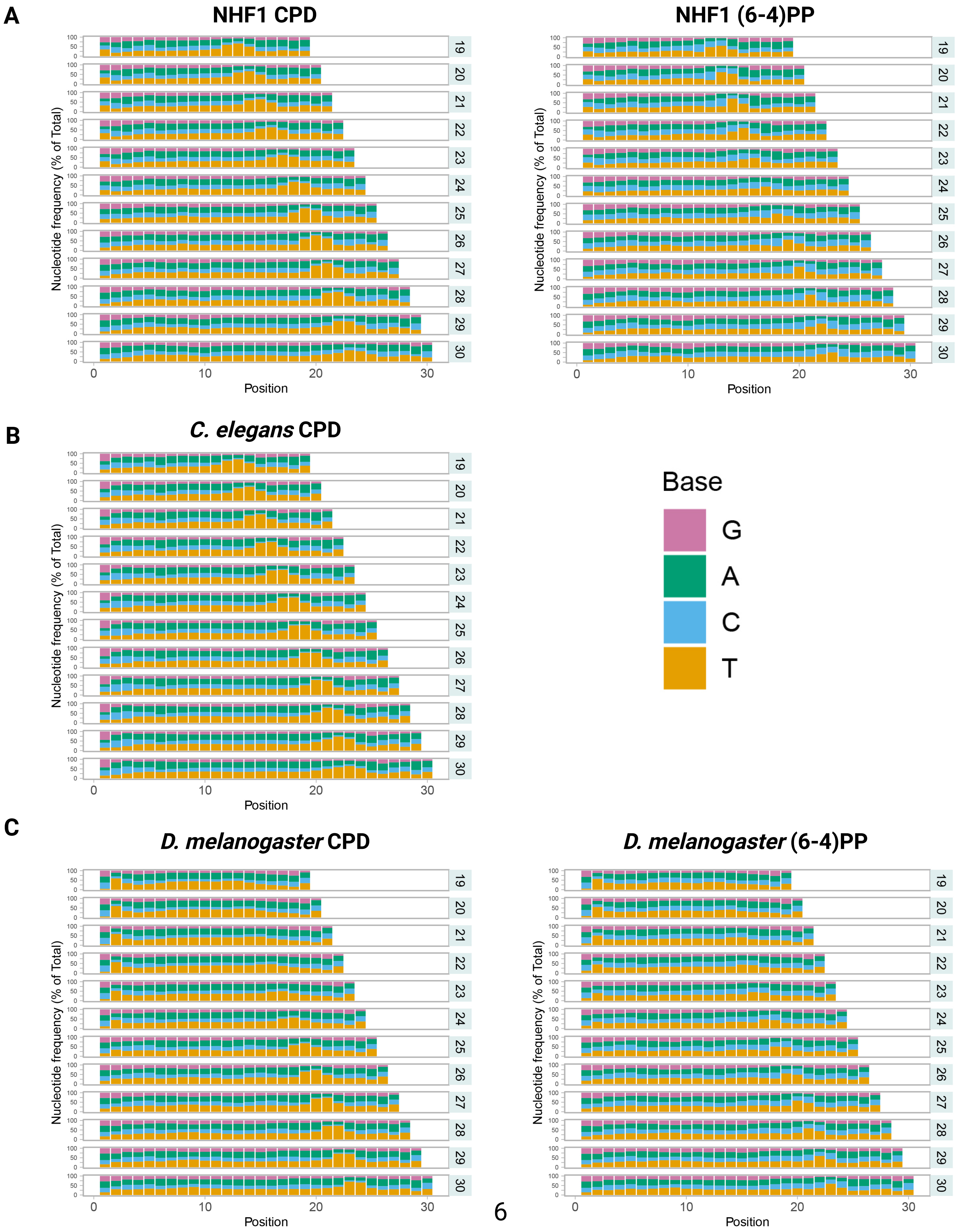

Figure S4. Analysis of nucleotide frequencies at each position along qXR-Seq reads of different lengths (19-30nt) in wildtype **(A)** NHF1 cells, **(B)** *C. elegans*, and **(C)** *Drosophila*. For downstream analysis we selected read lengths of 24-30nt, 20-28nt, and 25-30nt, respectively, because these had the greatest dipyrimidine enrichment from the expected distance from the 3’ end. Data from the CPD and (6-4)PP qXR-Seq experiments are shown on the left and right, respectively.

Supplemental Figure 5.

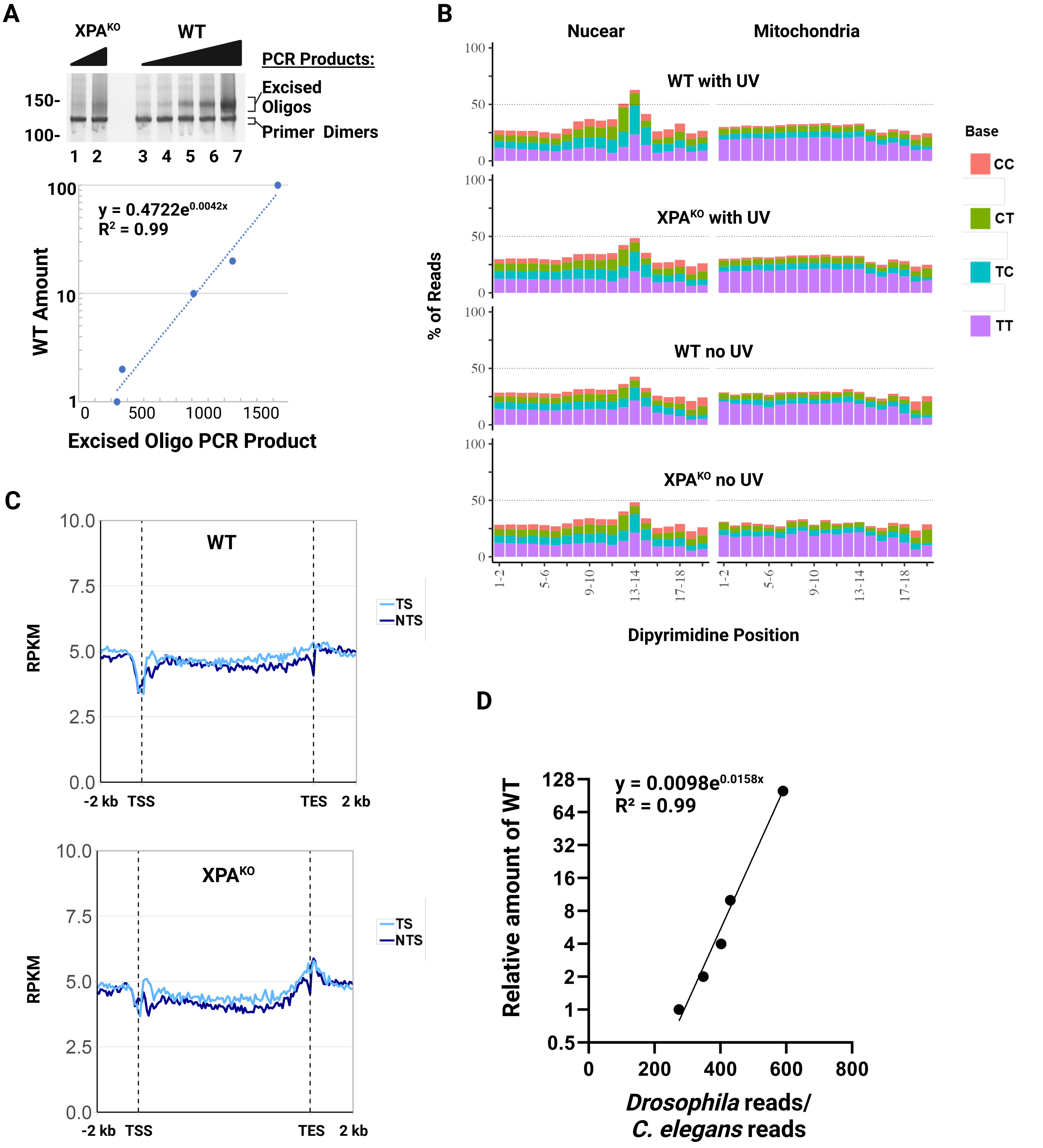

Figure S5. (6-4)PP qXR-Seq indicates levels of excision repair in *Drosophila* lacking XPA that are similar to CPD. (A) Analysis of dsDNA libraries of the excised (6-4)PP-containing oligos by polyacrylamide gel electrophoresis. Ligation products were PCR-amplified with fifteen cycles, and the PCR product descriptions are indicated on the right, sizes (base pairs) on the left, and quantitation of WT is shown below. **(B)** Analysis of the frequency of the possible dipyrimidines along (6-4)PP qXR-Seq reads of 25-30 nt length (trimmed to 20nt from the 5' end) from the indicated fly strains and UV conditions mapped to either nuclear genome DNA (left) or mitochondrial DNA (right). **(C)** Analysis of transcription-coupled repair in the WT (top) and XPA^{KO} (bottom) fly strains. (6-4)PP qXR-Seq data is plotted as average repair reads (y-axis) along the length of a "unit gene" (x-axis) as described in Figure 3G. **(D)** The spike-in analysis of the fly:worm read ratio from the UV-irradiated WT dilution was used to determine the percentage of excised (6-4)-containing oligos in the different samples relative to WT. There was approximately 0.85% (6-4)PP-containing excised oligos in the UV-irradiated XPA^{KO} flies relative to WT, which was 85-fold more than in either the unirradiated WT or XPA^{KO} flies, both were approximately 0.01% relative to UV-irradiated WT. RPKM, reads per kilobase per million mapped reads; TSS, transcription start site; TES, transcription end site. TS, transcribed strand; NTS, nontranscribed strand.
