# Supplementary material for: Cross-species investigation into the requirement of XPA for nucleotide excision repair
Source: Nucleic Acids Res. 2023 Nov 22;52(2):677–89. doi: 10.1093/nar/gkad1104 (PMC10810185; doi:10.1093/nar/gkad1104)
Supplement: gkad1104_Supplemental_Files [file gkad1104_supplemental_files.zip › Figure Legends for Supplemental Tables.docx]

**Supplemental Table S1. Distribution of XPA and XPC homologs among 2132 species.**The first column displays the species scientific names along with their corresponding taxonomic IDs. The second and third columns provide the counts within each species of XPA and XPC homologs, respectively.

**Supplemental Table S2**. List of XR-seq data with descriptive information about the samples.
